# Derivation of a novel risk assessment model of venous thromboembolism in hospitalized patients

**DOI:** 10.7717/peerj.21598

**Published:** 2026-08-04

**Authors:** Zhiliang Zhou, Jie Weng, Fan Fei, Yaqi Xu, Jiaze Song, Chen Liu, Wenyi Jin, Fengyu Chen, Liang Wang, Chan Chen, Zhiyi Wang, Zhe Xu

**Affiliations:** 1 General Practice, The Second Affiliated Hospital and Yuying Children’s Hospital of Wenzhou Medical University, Wenzhou, China; 2 Wenzhou Key Laboratory of Precision General Practice and Health Management, Wenzhou, China; 3 Institute of Hypoxia Medicine, School of Basic Medical Sciences, Wenzhou Medical University, Wenzhou, Zhejiang, China; 4 The Central Health Center of Sanshipu Town, Lu’an, China; 5 General Practice, Lu’an Hospital of Anhui Medical University, Lu’an, China; 6 Public Health, Marshall University, West Virginia, United States; 7 Geriatric Medicine, The First Affiliated Hospital of Wenzhou Medical University, Wenzhou, China; 8 General Practice, Beijing Tsinghua Changgung Hospital, School of Clinical Medicine, Tsinghua University, Beijing, China; 9 Intensive Care Unit, The Second Affiliated Hospital and Yuying Children’s Hospital of Wenzhou Medical University, Wenzhou, China

**Keywords:** Venous thromboembolism, Pulmonary embolism, Risk assessment model, Hospitalization, Inpatients

## Abstract

**Background:**

Venous thromboembolism (VTE), which includes deep vein thrombosis (DVT) and pulmonary embolism (PE), significantly contributes to morbidity and mortality among hospitalized patients. Despite the existence of various VTE risk assessment models (RAMs), their performance in accuracy, sensitivity and specificity remain suboptimal, highlighting opportunities to improve predictive accuracy for clinical decision-making.

**Methods:**

We conducted a retrospective multicenter study involving three hospitals, which enrolled patients with VTE from January 1, 2021, to December 30, 2023. A novel RAM (Weng score) was developed through three different strategies: clinical knowledge-driven model (Model A), data-driven model (Model B), and decision tree-based model (Model C). The primary outcome was in-hospital VTE. Prediction of PE alone was examined as a secondary outcome. Model performance was evaluated through discrimination, calibration, precision, and decision curve analysis (DCA).

**Results:**

A total of 1,791 patients were analyzed, with 680 VTE events recorded during hospitalization. The Weng score, derived from Model A, demonstrated superior predictive performance for VTE and PE compared to existing RAMs, with an area under the receiver operating characteristic curve (AUROC) of 0.895 (95% confidence interval (CI) [0.880–0.909]) for VTE and 0.877 (95% CI [0.851–0.903]) for PE. In comparison, the AUROCs for existing RAMs (Caprini, Padua, Wells, Geneva, and Autar scores) ranged from 0.687 to 0.789 for VTE prediction and from 0.682 to 0.769 for PE prediction. The Weng score also demonstrated excellent calibration and discrimination, outperforming the Caprini, Padua, Wells, Geneva, and Autar scores in hospitalized patients. The Weng score’s clinical utility for relative risk stratification was further supported by DCA within this case-control sampled cohort, showing a higher net benefit in predicting VTE and PE than existing RAMs.

**Conclusions:**

We developed and internally validated the Weng score using retrospective data from Chinese hospitals. While it showed more favorable calibration and discrimination than existing RAMs in our cohort, external validation in diverse settings and prospective studies accounting for anticoagulation management are essential before clinical adoption. The Weng score is intended for VTE risk stratification only; clinical decisions regarding thromboprophylaxis should integrate both VTE and bleeding risk assessments using validated tools. Because the current model was derived from a case-control sampled cohort (oversampled VTE events), the absolute risk estimates and net benefit findings reflect relative risk ranking and require external calibration in a representative prospective cohort before any clinical implementation.

## Introduction

Venous thromboembolism (VTE), which includes deep vein thrombosis (DVT) and pulmonary embolism (PE), is a major contributor to morbidity and mortality among hospitalized patients (Hunt, 2009). DVT is defined as a blood clot (thrombus) within the deep veins, typically in the legs (*e.g*., calf or thigh) or pelvis. PE is a sudden blockage of one or more pulmonary arteries, most often caused by a thrombus that travels from a DVT. The prevalence of VTE among hospitalized patients varies from 4.96% to 14.9% in clinical trial populations (Samama et al., 1999; Cohen et al., 2006), with a 30-day case-fatality rate for PE ranging from 5% to 15% in epidemiological studies (Barco et al., 2020; Naess et al., 2007). While VTE is particularly common among patients who have undergone surgery (Falck-Ytter et al., 2012), approximately 75% of hospital-acquired VTE cases occur in medical inpatients (Goldhaber, Dunn & MacDougall, 2000). VTE is a potentially preventable disease, and appropriate preventive measures can be implemented for high-risk groups to reduce VTE (Samama et al., 1999; Cohen et al., 2006; Leizorovicz et al., 2004). The prevention of VTE is predicated on the accurate identification of patients at increased risk, necessitating the use of VTE risk assessment models (RAMs) (Kearon et al., 2012).

Existing RAMs, such as the Caprini (Caprini, 2010) (originally developed for surgical patients), Padua (Barbar et al., 2010), Autar (Autar, 1996), Geneva (Le Gal et al., 2006), Wells (Wells et al., 2001), and IMPROVE scores (Spyropoulos et al., 2011), were developed to stratify VTE risk among inpatients and guide the prescription of prophylactic anticoagulation. However, these RAMs have exhibited varying degrees of sensitivity and specificity, with some showing limited discriminative power when evaluated through the area under the receiver operating characteristic curve (AUROC) (Blondon et al., 2018). Recently, a multicenter prospective cohort study has reported suboptimal accuracy and prognostic performance of these RAMs (Häfliger et al., 2024). This raises concerns regarding their clinical utility and predictive accuracy for VTE.

While comprehensive clinical evaluation remains paramount, improving the accuracy of VTE risk assessment tools could enhance decision-making. Current limitations of established RAMs suggest value in developing better-performing models applicable across inpatient settings. A new model should integrate both clinical and laboratory parameters that are readily available upon admission, thereby facilitating timely and targeted thromboprophylactic interventions. This research intends to create an innovative risk assessment model for hospital-acquired VTE in hospitalized patients, utilizing a comprehensive set of objective and clinically relevant risk factors. The study population consists of hospitalized patients, and the primary outcome is VTE events occurring during the hospital stay. By employing robust statistical methods, we seek to enhance the predictive accuracy and clinical applicability of VTE risk assessment. The goal is to enhance the predictive capabilities for VTE, enabling a more personalized approach to patient care and ultimately reducing the incidence of this potentially life-threatening condition.

## Materials and Methods

### Study design and participants

This retrospective multicenter study was conducted across three tertiary hospitals in China (The Second Affiliated Hospital of Wenzhou Medical University; The First Affiliated Hospital, Wenzhou Medical University; Lu’an Hospital of Anhui Medical University) between 1 January 2021 and 30 December 2023. Patient data were extracted from electronic medical records in general medicine wards (internal medicine, geriatrics), general surgery departments (including orthopedic surgery), and intensive care units (ICU). Study participants were patients aged ≥18 years discharged from three hospitals. We employed a case-control sampling design within the overall inpatient cohort. All eligible patients who developed VTE during hospitalization were identified, and non-VTE controls were randomly selected from the same hospitalization periods at an approximate 1:2 ratio to ensure adequate representation of the non-event population while maintaining feasibility of data extraction. We defined venous thromboembolism as a diagnosis of “DVT”, “deep vein thrombosis”, “PE” or “pulmonary embolism” in hospital or discharge records. For patients who were hospitalized multiple times during the study period, only data from the first hospitalization were assessed. This avoided clustering of events within the same individual. Accordingly, if a patient developed VTE during a subsequent hospitalization, they were not included as a case unless VTE occurred during their first hospitalization. Exclusion criteria were as follows: primary diagnosis of VTE or VTE-related complications on admission, isolated superficial VTE, post-resuscitation status, age < 18 years, hospital stay <3 days, and prior anticoagulant therapy. The primary outcome was in-hospital occurrence of VTE (a composite of DVT and PE). Secondary analysis focused on PE alone. DVT was diagnosed on the basis of confirmation of thrombus in the deep veins of the legs, deep pelvic veins, or vena cava by venous duplex ultrasonography or computed tomography (CT) scanning (Konstantinides et al., 2020). PE was confirmed by multidetector computed tomographic pulmonary angiography (CTPA) (Konstantinides et al., 2020). This study was conducted in accordance with the ethical standards of the responsible committees on human experimentation and with the Helsinki Declaration of 1975. The study involved retrospective analysis of de-identified patient data; therefore, the Ethics Committee of the Second Affiliated Hospital and Yuying Children’s Hospital of Wenzhou Medical University approved a waiver of informed consent (Ethical Application Ref: IRB# 2022-K-36-01), consistent with Chinese regulations and ethical guidelines for retrospective research using anonymized data.

### Patient identification and data collection

We used two methods to identify potentially eligible patients. First, the built-in diagnostic search function of the medical record system (based on ICD-10 codes) was used to identify all inpatient cases with admission and discharge diagnoses containing DVT and PE. The primary admission diagnosis was recorded separately from the discharge diagnosis, and only new diseases or diagnoses discovered during the hospital stay were recorded on the discharge diagnosis. The second method was a “keyword search”, which used structured query language of the big data retrieval system to search for specific terms in free-text fields of electronic discharge records and the Picture Archiving and Communication System (PACS). Search terms included “venous thrombosis”, “venous thromboembolism”, and “pulmonary embolism”. Both methods were used to maximize sensitivity, and overlapping cases were deduplicated. The search results generate a list of discharged patients containing at least one related term. Data from patients’ electronic medical records were collected manually and reviewed by two researchers. Discrepancies between the two reviewers were resolved by consensus with a third senior researcher.

### Sample size

Based on the events-per-variable (EPV) principle for regression modeling, we targeted 5–10 EPV to minimize overfitting, with 10 EPV considered ideal. This required 50–100 VTE events to evaluate 10 potential predictors. A total of 66 candidate variables were initially collected. With 680 VTE events identified, we could reasonably evaluate up to 68 predictors in the final model. To ensure robustness, we sought >600 confirmed VTE events. During retrospective screening, we identified potential VTE patients through diagnostic coding and keyword searches, then applied a 1:2 sampling ratio (VTE: non-VTE) from the same hospitalization periods. Therefore, we reviewed 2,500 patient records to obtain sufficient qualifying cases, yielding a final case-control sampled cohort of 680 VTE events and 1,111 controls (1:1.6 ratio) after applying exclusion criteria. The resulting 38% VTE proportion in the final sample reflects this sampling strategy and does not represent the true population incidence of hospital-acquired VTE.

### Variables

Sixty-six candidate variables were collected (Table 1), including five demographic variables, 14 comorbidities, five vital signs, four symptomatic signs, four medication histories, five trauma-related variables, nine other categories, and 20 laboratory test variables. Age was stratified in 10-year increments. Body mass index (BMI) is categorized in accordance with the World Health Organization’s criteria. Heart rate groupings were based on the Wells scale. Recent trauma and surgery, as well as absolute bed rest exceeding 3 days, were categorized according to the Padua scale. Other variables, such as laboratory tests, were grouped based on clinical practice, ease of use, or median values (ejection fraction values).

**Table 1:** Candidate variables.

Domains	Variables*
Demographics	Age†, Male, BMI, smoking, drinking
Chronic/acute comorbidities	Diabetes mellitus, hypertension, chronic heart disease, atrial fibrillation†, acute myocardial infarction†, angiosclerosis†, cancer†, diabetes insipidus, autoimmune disease†, nephrotic syndrome†, inflammatory bowel disease†, thrombophilia†, sepsis†, stroke†
Vital signs on admission	Temperature, respiratory rates, systolic blood pressure, diastolic blood pressure, heart rate†
Symptoms	Paralysis†, lower limb swelling†, varicose veins†, pleural effusion†
Medication history	Glucocorticoid†, contraceptive drug, dehydrant, immunosuppressive drug†
Surgery-related	History of surgery ≤1 month†, history of trauma ≤1 month†, artificial instrument implantation (such as breast implants, fracture nails), cardiac pacemaker, general anesthesia
Others	VTE history†, unexplained habitual abortion, pregnancy or puerperium, strict bed rest >3 days†, central venous catheter, mechanical ventilation, hypertonic dehydration, ICU admission, EF
Laboratory tests	CRP, WBC, ANC, ALC, Hb, HCT, PLT, ALT, AST, TP, Alb†, TBIL, Cr, Ca, TG, TC, LDL, HDL, D-Dimer†, FIB

**Notes:**

* All variables were included in statistics-driven and decision tree model.

† Variables selected for the clinical knowledge-driven model.

BMI, Body mass index; VTE, venous thromboembolism; CRP, C-reactive protein; WBC, white blood cell; ANC, absolute neutrophil counts; ALC, absolute lymphocyte counts; Hb, hemoglobin; HCT, hematocrit; PLT, platelet count; ALT, alanine transaminase; AST, aspartate aminotransferase; TP, total protein; Alb, albumin; TBIL, total bilirubin; Cr, creatinine; Ca, calcium; TG, triglyceride; TC, total cholesterol; LDL, low-density lipoprotein; HDL, high-density lipoprotein; FIB, fibrinogen; EF, ejection fraction.

### Missing data

Missing data only included BMI 61 (3.41%), Hemoglobin A1c 56 (3.1%), and serum calcium 43 (2.40%). Multiple imputation with five iterations was performed using the MICE package in R to complete data filling.

### Model development

To ensure robustness and effectiveness, three different strategies were used to develop predictive models: clinical model based on knowledge (Model A), data-driven model (Model B), and decision tree-based model (Model C).

**Model A:** The selection of predictor variables was based on (1) existing literature reports on VTE risk factors and (2) expert clinical experience with VTE. The variables finally included in the model were all variables strongly suspected to be associated with VTE in clinical practice. Before using the standard logistic regression model, to avoid overfitting, we set the EPV to 10 as the ideal ratio (Vittinghoff & McCulloch, 2007). We entered all preselected predictor variables into regression model, ultimately retaining variables with a *p*-value less than 0.05.

**Model B:** This model examines predictor variables based solely on statistical analysis of the data without considering the importance of each variable. This exploratory statistical approach aims to identify new and potential predictor variables that may not have been reported previously. Considering the power of variable selection, the least absolute shrinkage selection operator (LASSO) method was used to identify potential variables (Steyerberg et al., 2000). Penalized regression performs variable selection by constraining the regression coefficients using a penalty coefficient (λ) (Pavlou et al., 2015). Variables selected from the LASSO regression were subsequently entered into a multivariable logistic regression model. Only predictors with a *P* value < 0.05 were retained as independent risk factors in the final Model B. This approach ensured that all variables in the final model had statistically significant associations with VTE.

**Model C:** A decision tree classifier was constructed using the C5.0 algorithm. This non-parametric method, which doesn’t assume any statistical distribution of the data, was deemed suitable for VTE prediction model (Friedl & Brodley, 1997). The C5.0 decision tree algorithm uses information gain rate to select the best variable for splitting, forming a tree structure. It employs post-pruning from the leaf nodes upwards, focusing on error estimation and pruning criteria (Freund & Schapire, 1997). Key parameters were set as follows: minimum cases per leaf = 10, pruning severity = 75%, and number of boosting trials = 10. The resulting tree structure provides an interpretable set of decision rules for VTE risk classification.

### Model performance

We evaluated model performance through discrimination, calibration, and precision assessments (Steyerberg et al., 2010). The AUROC was used to test the model’s ability to identify VTE, and the calibration curve and Hosmer-Lemeshow (H-L) fit test were used to calibrate the model. Furthermore, we used the non-parametric DeLong method to compare two AUROC values of the same sample size (DeLong, DeLong & Clarke-Pearson, 1988). The H-L fit test and calibration plots are used to evaluate the calibration of the model, whereas the sensitivity of the H-L fit test is limited by the sample size, which groups patients into deciles regardless of individual risk levels (Kramer & Zimmerman, 2007; Bertolini et al., 2000). In addition, the calibration curves derived from the H-L test are not true curves. To address the limitations, the GiViTI calibration belt was introduced (Serrano, 2012; Nattino, Finazzi & Bertolini, 2014). This method uses a polynomial logistic function to model the relationship between predicted and observed probabilities, generating a calibration curve with 80% and 95% confidence bands. A statistically significant deviation from perfect calibration is noted if the 95% confidence band does not intersect the bisector (the line of perfect agreement). The GiViTI approach provides a formal calibration test that avoids arbitrary grouping of patients into deciles, offering a more robust assessment of model fit than the traditional H-L test. The prediction model’s overall performance, measured by precision, was assessed using the Brier score, which reflects the average squared difference between observed within sample and sample-based predicted VTE, encompassing both discrimination and calibration. Brier scores range from 0 to 1, with lower scores indicating better model performance.

### Model validation

Internal validation was performed using bootstrapping with 1,000 resamples to provide optimism-corrected performance estimates. For each bootstrap sample, the full modeling process—including predictor pre-selection (for Model A), LASSO variable selection (Model B), decision-tree construction (Model C), and logistic regression coefficient estimation—was repeated independently. The resulting models were then applied to the original development dataset to calculate optimism-corrected discrimination, calibration, and Brier scores. This approach follows TRIPOD recommendations for datasets with a moderate number of events when data splitting is not optimal. Although the dataset contains 680 VTE events, a 75/25 split would reduce the development sample to approximately 510 events (with only ~170 VTE events in the validation set), which could lead to unstable coefficient estimates and overfitting. Bootstrap resampling is a widely accepted method for internal validation in prediction modeling studies and is explicitly recommended by TRIPOD guidelines when splitting is not optimal. The optimism-corrected AUROC and calibration metrics were derived by averaging the differences between bootstrap performance and original model performance.

### Development risk score

This study devised the Weng scoring system to predict VTE. The Weng score was derived by assigning each predictor an integer or half-integer weight proportional to its regression coefficient relative to the smallest coefficient in the model. The aggregate of these assigned scores constituted the total score for each individual, which was subsequently incorporated into the final regression model.

### Decision curve analysis

To assess the clinical utility of the Weng score in predicting VTE, decision curve analysis (DCA) was conducted to compare the net benefits of the Weng, Caprini, Padua, Autar, Geneva, and Wells scores across various threshold probabilities. DCA evaluates clinical utility by quantifying ‘net benefit’ (true positives minus harms from false positives) across risk thresholds. This helps clinicians weigh trade-offs when using RAMs for prophylaxis decisions.

### Definition of analytic samples for VTE and PE prediction

For VTE prediction, the full cohort of 1,791 patients (680 VTE cases, 1,111 non-VTE controls) was used. For PE prediction, the analysis was restricted to 163 PE cases and the same 1,111 non-VTE controls (total *n* = 1,274), excluding the 517 patients with DVT only. This sampling strategy was chosen to ensure that the PE model specifically discriminates between patients with PE and those without any VTE, without dilution by DVT-only patients. Consequently, the predicted probabilities for PE from this case-control derived sample reflect relative risk ordering rather than absolute population risks. Calibration and net benefit estimates for PE should be interpreted with this sampling design in mind, and external validation in a prospective cohort is required to obtain absolute risk estimates.

Statistical analysis was performed using R version 4.4.0 (R Core Team, 2024). For internal validation, bootstrap resampling with 1,000 iterations was used to obtain optimism-corrected performance estimates, consistent with TRIPOD guidelines for prediction model development when sample splitting is not optimal.

## Results

### Cohort

**Figure 1:**
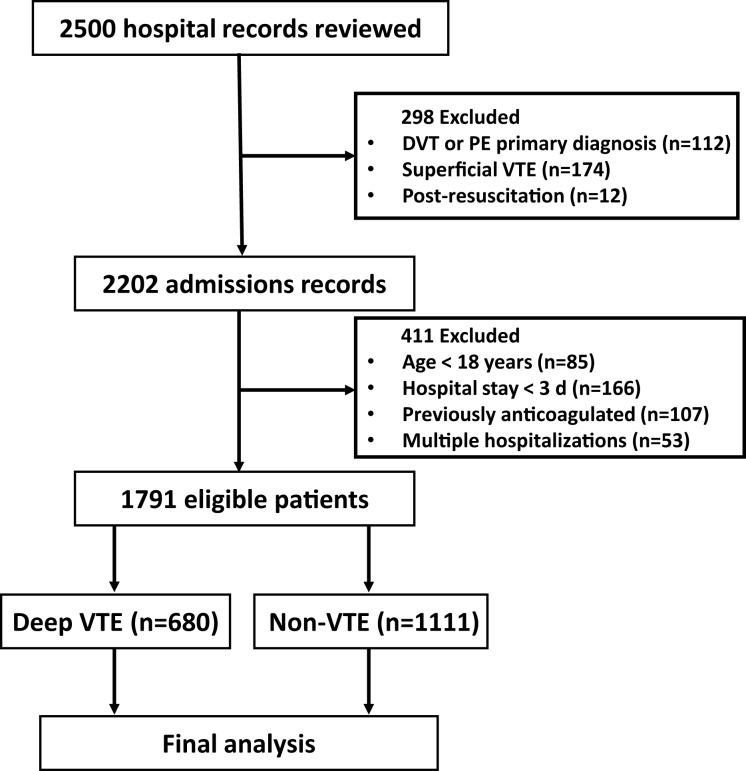
The study flow diagram.

In the final analysis, a total of 1,791 patients were included in the study. Of these, 680 cases (38%) experienced VTE events during their hospital stay. Among the 680 VTE patients, 163 individuals (24%) further developed PE. Patients who developed VTE during hospitalization exhibited significant differences in various aspects, including age, chronic/acute comorbidities, vital signs, symptoms, medication history, surgery-related factors, and laboratory tests. The study flow diagram and cohort characteristics are displayed in Fig. 1 and Table S1.

### Prediction models

These three approaches identified different variables associated with VTE, as detailed in Table 2. Model A first preselected 24 candidate variables on the basis of clinical knowledge (Table 1), and ultimately identified 14 variables strongly associated with VTE (Table 2). In the data-driven model B, LASSO regression analysis (Fig. S1) initially selected 28 candidate predictors. These were then entered into a multivariable logistic regression model, and only predictors with *P* < 0.05 were retained. This process yielded 15 independent predictors (Table S2), of which 14 corresponded to variables in Model A (Table 2; Table S2). Model C used a decision tree approach and yielded five significant predictors. Three of these variables were consistent across all three models: ‘VTE history’, ‘strict bed rest > 3 days’ and ‘D-dimer > 0.5’.

**Table 2:** Three approaches for model development and their performance.

Approaches	No. of variables	Variables	Discrimination		Calibration		Precision
			AUROC	95% CI	H-L/*P*-value	GiViTI *P*-value	Brier score
**Model A**: Clinical knowledge-driven	14	Age, cancer, stroke, thrombophilia, heart rate on admission >100 bpm, paralysis, lower limb swelling, varicose veins, pleural effusion, history of trauma ≤1 month, VTE history*, strict bed rest >3 days*, Alb <40 g/L, D-Dimer*	0.899	[0.885–0.913]	5.451/0.709	0.339	0.125
**Model B**: Data-driven	15	Age, cancer, stroke, thrombophilia, heart rate on admission >100 bpm, paralysis, lower limb swelling, varicose veins, pleural effusion, history of trauma ≤1 month, VTE history*, Strict bed rest >3 days*, Alb <40 g/L, D-Dimer*, TG >1.7 mmol/L	0.900	[0.885–0.914]	5.262/0.729	0.310	0.124
**Model C**: Decision tree	5	VTE history*, strict bed rest >3 days*, central venous catheter, CRP, D-Dimer*	0.832	[0.814–0.851]	15.953/0.043	0.100	0.153

**Notes:**

* Selected by all three approaches.

H-L, Hosmer-Lemeshow test; GiViTI, Italian group for the evaluation of intervention in intensive care medicine; VTE, venous thromboembolism; Alb, albumin; bpm, beats per minute; TG, triglyceride; CRP, C-reactive protein.

For model performance, the AUROC for Model A and Model B was 0.899 (95% CI [0.885–0.913]) and 0.900 (95% CI [0.885–0.914]), respectively, superior to Model C’s 0.832 (95% CI [0.814–0.851]). In terms of model calibration, models A and B were (chi-square = 5.451, *P* = 0.709) and (chi-square = 5.262, *P* = 0.729) better than model C (chi-square = 15.953, *P* = 0.043), respectively, by the Hosmer-Lemeshow test (Table 2). GiViTI correction bands showed no significant deviation (*P* > 0.05) from the bisector for all prediction models (Table 2, Fig. S2). The accuracy (brier score) of models A and B is similar, and both are better than model C.

Internal validation was performed by resampling 1,000 times. The optimism-corrected AUROCs of Model A and Model B were 0.881 (95% CI [0.870–0.895]) and 0.886 (95% CI [0.872–0.933]), respectively, which were similar to the original apparent AUROCs (0.899 and 0.900), indicating minimal overfitting. Considering that models A and B are significantly better than model C, as well as the adaptability and simplicity of model A, we finally selected the knowledge-based clinical model as the final model. Model A and Model B showed nearly identical discrimination, with AUROCs of 0.899 and 0.900, respectively. The difference was not statistically significant (DeLong test, *P* = 0.78). Both models also demonstrated good calibration and same precision. Given their equivalent predictive performance, we selected Model A as the basis for the Weng score for the following objective reasons: (1) parsimony—Model A includes 14 predictors, whereas Model B includes 15 (the additional variable is triglyceride, which did not materially improve performance); (2) clinical interpretability—all variables in Model A are routinely available at admission and have well-established associations with VTE, whereas the added variable in Model B (triglyceride) has a less established and weaker biological link to VTE in hospitalized patients. Thus, Model A was chosen for the development of the Weng score.

### Weng risk score

Based on the predictor variables identified above, we created a new VTE risk score (Weng score) and assigned a score to each variable based on its regression coefficient (Table 3). The Weng risk score of the patient was calculated by summing the scores of the individual items. The total Weng score ranged from 0 to 70.5. For example, a patient aged >70 years (+4.5 points), with a history of VTE (+11.5 points), D-dimer >0.5 μg/mL (+7.0 points), and strict bed rest >3 days (+3.0 points) would have a total score of 26.0. We developed a simple article-based checklist with the scoring table for clinical use.

**Table 3:** Variables in the final model using multivariable regression.

Variable	OR (95% CI)	*P*-value	Coefficients	Point‡
Age, years				
≤30	–	–	–	–
>30 to ≤40	1.13 [0.34–3.72]	0.839	0.123	0
>40 to ≤50	1.12 [0.38–3.28]	0.831	0.117	0
>50 to ≤60	2.35 [0.84–6.57]	0.103	0.855	0
>60 to ≤70	3.01 [1.09–8.30]	0.033	1.103	3.5
>70	3.72 [1.37–10.13]	0.010	1.313	4.5
Cancer	2.11 [1.47–3.02]	<0.001	0.746	2.5
Stroke	2.33 [1.49–3.65]	<0.001	0.847	3.0
Thrombophilia	43.69 [9.02–211.57]	<0.001	3.777	12.5
HR >100 bpm	3.86 [2.09–7.13]	<0.001	1.350	4.5
Paralysis	4.00 [1.31–12.21]	0.015	1.387	4.5
Lower limb swelling	4.51 [2.78–7.32]	<0.001	1.506	5.0
Varicose veins	3.64 [1.71–7.71]	0.001	1.291	4.5
Pleural effusion	1.79 [1.26–2.53]	0.001	0.581	2.0
Trauma history	4.77 [3.30–6.90]	<0.001	1.563	5.0
VTE history	33.12 [13.02–84.26]	<0.001	3.500	11.5
Strict bed rest >3 days	2.41 [1.80–3.22]	<0.001	0.878	3.0
Alb <40 g/L	1.35 [1.01–1.81]	0.045	0.301	1.0
D-Dimer >0.5 μg/mL	8.26 [5.35–12.75]	<0.001	2.111	7.0
Total score				0–70.5

**Notes:**

‡ Assignment of points to risk factors was based on a linear transformation of the corresponding β regression coefficient. The coefficient of each variable was divided by 0.301 (the smallest absolute β value, corresponding to Alb<40 g/L) and allocated an integer or an half integer score for each variable.

HR, Heart rate; VTE, venous thromboembolism; Alb, albumin; OR, odds ratio; CI, confidence interval.

### Comparison of Weng score and clinical RAMs in predicting VTE

The performance of the Weng score in predicting VTE in hospitalized patients was compared with the Caprini, Padua, Wells, Geneva and Autar scores. The AUROC for the Weng score was 0.895 (95% CI [0.880–0.909]), which was significantly higher than that for the Caprini 0.698 (95% CI [0.674–0.723]), Padua 0. 786 (95% CI [0.765–0.808]), Wells 0.678 (95% CI [0.663–0.710]), Geneva 0.716 (95% CI [0.629–0.739]) and Autar 0.789 (95% CI [0.768–0.810]) (Table 4; Fig. S3A; Table S3). Similarly, in the medical and surgical populations, the Weng score predicted VTE with AUROC of 0.900 (95% CI [0.881–0.918]) and 0.885 (95% CI [0.861–0.909]), respectively, both significantly higher than the Caprini, Padua, Wells, Geneva and Autar scores (Table 4; Figs. S3B, S3C; Tables S4, S5). Therefore, the results demonstrated that the Weng score showed higher discrimination for predicting thromboembolism than the other clinical VTE RAMs in this cohort. In addition, the accuracy of Weng scores was assessed using the H–L Chi-square test (Table 4), calibration curves (Figs. S4–S6) and GiViTI calibration bands (Fig. S7) for the total inpatient, internal medicine and surgical populations. All three tests demonstrated that the Weng score, in both medical and surgical inpatients, had favorable calibration.

**Table 4:** Performance comparison of six scores for predicting thromboembolism.

	All population (*n* = 1,791)				Internal medicine population (*n* = 1,070)				Surgical population (*n* = 721)			
Score	AUROC	*P*-value	H-L	*P*-value	AUROC	*P*-value	H-L	*P*-value	AUROC	*P*-value	H-L	*P*-value
Caprini score	0.698 (0.674–0.723)	<0.001	28.747	<0.001	0.717 (0.685–0.749)	<0.001	49.997	<0.001	0.654 (0.614–0.694)	<0.001	13.931	0.030
Padua score	0.786 (0.765–0.808)	<0.001	38.429	<0.001	0.808 (0.780–0.835)	<0.001	15.018	0.010	0.745 (0.708–0.782)	<0.001	65.523	<0.001
Wells score	0.687 (0.663–0.710)	<0.001	0.564	0.453	0.746 (0.716–0.775)	<0.001	3.878	0.049	0.569 (0.532–0.606)	<0.001	9.049	0.003
Geneva score	0.716 (0.692–0.739)	<0.001	0.458	0.796	0.748 (0.719–0.777)	<0.001	4.265	0.119	0.658 (0.620–0.696)	<0.001	5.739	0.057
Autar score	0.789 (0.768–0.810)	<0.001	10.681	0.220	0.808 (0.781–0.835)	<0.001	8.755	0.363	0.745 (0.709–0.780)	<0.001	14.517	0.069
Weng score	0.895 (0.880–0.909)	–	5.378	0.614	0.900 (0.881–0.918)	–	7.270	0.401	0.885 (0.861–0.909)	–	5.004	0.660

**Notes:**

*P*-values are from DeLong’s method comparing the AUROC of each respective risk assessment model (RAM) with that of the Weng score as the reference. The Weng score row shows ‘–’ because it is the reference.

VTE, Venous thromboembolism; AUROC, area under the receiver operating characteristic curve; H-L, Hosmer-Lemeshow.

### Comparison of Weng score and clinical RAMs in predicting PE

**Figure 2:**
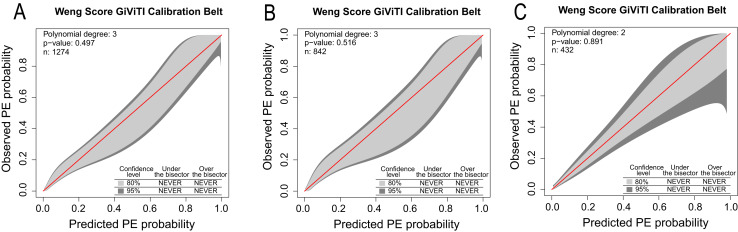
GiViTI calibration belts for the Weng score (derived from Model A) when predicting PE. (A–C) represent calibration of Weng score to predict PE in all patients, internal medicine patients and surgical patients. The predicted probabilities shown are derived from a case-control sampled cohort and reflect relative risk ordering; external calibration is needed to obtain absolute risk estimates.

Compared to predicting thromboembolism, the Weng score predicted PE with a slight decrease in discrimination, with an AUROC of 0.877 (95% CI [0.851–0.903]), which was still significantly higher than the Caprini 0.682 (95% CI [0.640–0.725]), Padua 0.769 (95% CI [0.729–0.809]), Wells 0.743 (95% CI [0.701–0.786]), Geneva 0.682 (95% CI [0.642–0.721]), and Autar 0.761 (95% CI [0.721–0.800]) (Table 5; Fig. S3D; Table S6). Similarly, in the medical and surgical groups, the Weng score demonstrated an ability to predict PE with an AUROC of 0.890 (95% CI [0.862–0.918]) and 0.850 (95% CI [0.790–0.910]), respectively (Tables S7, S8). These values were notably superior to those obtained from the Caprini, Padua, Wells, Geneva, and Autar scores (Table 5; Figs. S3E, S3F; Tables S6–S8). This suggests that the Weng score also demonstrated favorable predictive ability for PE relative to other RAMs in this cohort. In terms of accuracy, the H-L Chi-square test (Table 5), calibration curves (Figs. S8–S10), and GiViTI calibration bands (Fig. 2; Fig. S11) all showed that the Weng score had favorable calibration for accuracy in either medical patients or surgical patients.

**Table 5:** Performance comparison of six scores for predicting PE.

	All population (*n* = 1,274)				Internal medicine population (*n* = 842)				Surgical population (*n* = 432)			
Score	AUROC	*P*-value	H-L	*P*-value	AUROC	*P*-value	H-L	*P*-value	AUROC	*P*-value	H-L	*P*-value
Caprini score	0.682 (0.640–0.725)	<0.001	31.634	<0.001	0.699 (0.651–0.747)	<0.001	21.443	<0.001	0.730 (0.642–0.818)	<0.001	8.513	0.130
Padua score	0.769 (0.729–0.809)	<0.001	4.179	0.382	0.786 (0.743–0.830)	<0.001	4.813	0.307	0.792 (0.718–0.867)	<0.001	11.525	0.021
Wells score	0.743 (0.701–0.786)	<0.001	2.881	0.090	0.779 (0.734–0.824)	<0.001	1.501	0.2121	0.743 (0.643–0.843)	<0.001	5.565	0.018
Geneva score	0.682 (0.642–0.721)	<0.001	3.861	0.145	0.721 (0.677–0.764)	<0.001	3.049	0.217	0.610 (0.523–0.696)	<0.001	2.101	0.350
Autar score	0.761 (0.721–0.800)	<0.001	8.281	0.407	0.769 (0.725–0.813)	<0.001	7.345	0.394	0.841 (0.772–0.910)	<0.001	13.437	0.098
Weng score	0.877 (0.851–0.903)	–	7.553	0.374	0.890 (0.862–0.918)	–	8.475	0.293	0.850 (0.790–0.910)	–	10.56	0.103

**Notes:**

For PE prediction, the analysis included 163 PE cases and 1,111 non-VTE controls (total *n* = 1,274); patients with DVT only (*n* = 517) were excluded.

For PE prediction in the internal medicine population, the analysis included 124 PE cases and 718 non-VTE controls (total *n* = 842). Patients with DVT only were excluded from the PE-specific analyses. For PE prediction in the surgical population, the analysis included 39 PE cases and 393 non-VTE controls (total *n* = 432). Patients with DVT only were excluded from the PE-specific analyses.

*P*-values are from DeLong’s method comparing the AUROC of each respective RAM with that of the Weng score as the reference. The Weng score row shows ‘–’ because it is the reference.

PE, Pulmonary embolism; AUROC, area under the receiver operating characteristic curve; H-L, Hosmer-Lemeshow.

### Risk stratification

The risk stratification was based on the Weng score calculation method described in the Methods section (Development risk score section). The stratification of inpatient VTE risk is categorized into quartiles according to the Weng score: low risk (0 to 4), moderate risk (>4 to ≤10), high risk (>10 to ≤14), and very high risk (>14) (Table 6). Therefore, 462 (25.8%), 515 (28.75%), 386 (21.55%) and 428 (23.90%) patients were assigned to each of the four groups for the risk prediction of thromboembolism. Accordingly, the estimated VTE proportions in the four risk strata were 1.95%, 19.03%, 54.84% and 85.05% within this case-control sampled cohort. Within this case-control sampled cohort, the predicted VTE proportions showed good agreement with the observed event rates across the four risk strata (Table 6). These estimates are conditional on the sampling design (38% VTE prevalence in the sample) and reflect relative risk ordering rather than directly generalizable absolute population risks. True absolute risk calibration requires external validation in a prospective cohort with a representative event rate. Similar patterns were observed for PE prediction and across medical and surgical subgroups (Table 6). The Weng score is currently intended as a article-based research tool. No electronic calculator is required; users simply sum the point values from Table 3 and refer to the risk stratification in Table 6. Clinical adoption should await external validation in prospective cohorts.

**Table 6:** Risk of thromboembolism and pulmonary embolism according to risk stratification.

	Risk stratification	All population			Internal medicine population			Surgical population		
		*n* (%)	Sample-based predicted % (95% CI)	Observed % (within sample)	*n* (%)	Sample-based predicted % (95% CI)	Observed % (within sample)	*n* (%)	Sample-based predicted % (95% CI)	Observed % (within sample)
VTE	Low	462 (25.8)	2.00 [2.00–2.01]	1.95	294 (27.48)	1.04 [1.03–1.05]	1.02	168 (23.30)	3.76 [3.24–3.76]	3.57
	Moderate	515 (28.75)	19.00 [18.63–20.18]	19.03	342 (31.96)	17.19 [16.41–18.15]	17.25	173 (24.00)	22.55 [20.98–23.75]	22.54
	High	386 (21.55)	54.84 [53.00–55.14]	54.15	220 (20.56)	49.81 [49.79–52.35]	50.45	166 (23.02)	59.02 [57.66–60.38]	59.04
	Very high	428 (23.90)	85.27 [84.18–86.42]	85.05	214 (20.00)	84.70 [81.81–87.09]	83.64	214 (29.68)	86.36 [85.31–87.45]	86.45
PE	Low	455 (35.71)	0.44 [0.43–0.45]	0.44	292 (34.68)	0.34 [0.33–0.35]	0.34	163 (37.73)	0.66 [0.53–0.66]	0.61
	Moderate	452 (35.48)	7.60 [7.16–8.24]	7.74	310 (36.82)	8.56 [7.71–9.42]	9.25	142 (32.87)	5.63 [5.44–5.82]	5.63
	High	223 (17.50)	20.43 [20.05–21.19]	20.63	145 (17.22)	24.80 [23.38–25.32]	24.83	78 (18.06)	12.78 [12.45–13.03]	12.82
	Very high	144 (11.30)	56.30 [50.90–60.19]	55.56	95 (11.28)	63.39 [57.96–69.25]	63.16	49 (11.34)	39.16 [31.95–44.92]	40.82

**Notes:**

The risk category was calculated by adding the points for each of the following risk factors. The prognostic index was categorized in four groups: low risk (0–4 points), moderate risk (>4 to ≤10 points), high risk (>10 to ≤14 points), and very high risk (>14 points). For PE prediction, the analysis included 163 PE cases and 1,111 non-VTE controls (total *n* = 1,274); patients with DVT only (*n* = 517) were excluded. For PE prediction in the internal medicine population, the analysis included 124 PE cases and 718 non-VTE controls (total *n* = 842). Patients with DVT only were excluded from the PE-specific analyses. For PE prediction in the surgical population, the analysis included 39 PE cases and 393 non-VTE controls (total *n* = 432). Patients with DVT only were excluded from the PE-specific analyses. Sample-based predicted percentages were derived from the logistic regression model in this case-control sampled cohort. Observed percentages represent event rates within the same sample. Because the study used a case-control sampling design (38% VTE proportion in the sample *vs*. lower true population incidence), these percentages are conditional on the sampling strategy and should not be interpreted as directly generalizable absolute population risks. The Weng score is intended for relative risk stratification; external validation in a prospective representative cohort is required to calibrate absolute risk estimates for clinical use.

VTE, Venous thromboembolism; PE, pulmonary embolism; CI, confidence interval.

### Net benefit of the Weng score

**Figure 3:**
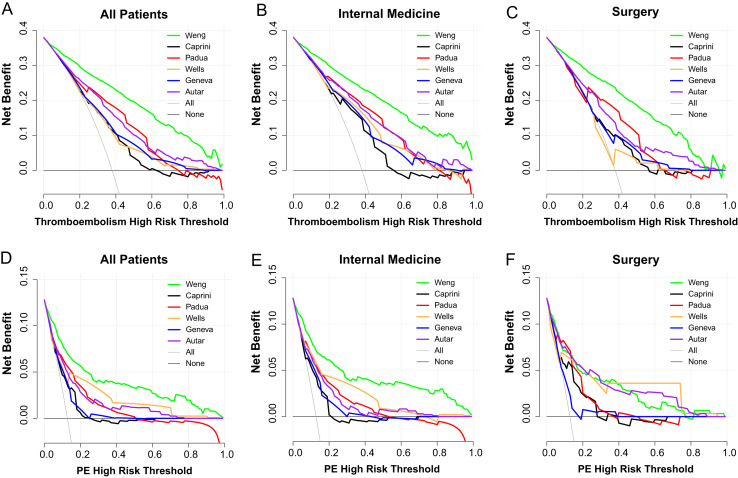
Decision curve analysis (DCA) for the Weng score (Model A) compared with existing RAMs. (A–C) represent the DCA curve of RAMs predicting VTE in all patients, internal medicine patients and surgical patients; (D–F) represent the DCA curve of RAMs predicting PE in all patients, internal medicine patients and surgical patients. Under the case-control sampling design, the predicted probabilities reflect relative risk ranking; the net benefit curves should be interpreted as supporting relative clinical utility, and absolute threshold probabilities require external validation.

Decision curve analysis (Fig. 3) showed that the Weng score provided a higher net benefit across a range of risk thresholds compared with existing RAMs, indicating superior relative clinical utility for risk stratification within this case-control sampled cohort. Because the predicted probabilities were derived from a case-control design (which oversampled VTE events) rather than a true incidence cohort, the net benefit curves should be interpreted as supporting relative risk ranking rather than providing directly actionable absolute treatment thresholds. External validation in a representative prospective cohort is needed to establish absolute net benefit for clinical decision-making.

## Discussion

In this retrospective, multicenter inpatient case-control sampled cohort study, we developed a new RAM, the Weng score, which incorporates risk factors for which data are known at the time of admission and can be easily obtained in clinical practice. The Weng score is designed to estimate VTE risk and support relative risk-based clinical decision-making within the context of this study, but it does not replace comprehensive clinical judgment-particularly regarding bleeding risk. When comparing the Weng score to published RAMs such as the Caprini, Padua, Wells, Geneva, and Autar scores, the Weng score showed enhanced performance, particularly in discrimination, sensitivity, and specificity. Furthermore, the Weng score demonstrated promising predictive capacity for PE in this cohort.

The risk of VTE in hospitalized patients has long been a major concern for healthcare providers. While several RAMs have been developed (Spyropoulos et al., 2011; Hayssen et al., 2022; Key et al., 2020; Ashrafi et al., 2022; Franchini et al., 2024; Trihan et al., 2021; Riondino et al., 2019; Streiff et al., 2021) and validated (Greene et al., 2016; Nendaz et al., 2014; Hostler et al., 2016). Previously released VTE RAMs had several limitations, including reliance on difficult-to-assess risk factors and a lack of validation or assessment in different populations (Darzi et al., 2020b, 2020a; Lyman et al., 2021). A systematic review by Pandor et al. (2021). found that the existing RAMs (Caprini, Padua, IMPROVE, Geneva and Kucher score) have generally weak predictive accuracy for VTE. There is insufficient evidence and too much heterogeneity to recommend the use of any particular RAMs. Prospective validation of the simplified and original Geneva, Padua and the IMPROVE by Häfliger et al. (2024) found suboptimal accuracy and prognostic performance in predicting VTE in internal medicine inpatients. However, the literature on RAMs that can be effectively used to predict DVT risk is sparse (Bo et al., 2020; Zhou et al., 2018). The available data suggest that the prediction accuracy of RAMs for VTE is generally poor, the evidence is insufficient, the heterogeneity is too strong to recommend the use of any particular RAMs and that more accurate VTE prediction strategies are needed (Häfliger et al., 2024; Pandor et al., 2021; Stuck et al., 2017; Moumneh et al., 2020).

Three different methods were used to build the model in present study, which allowed us to see the pattern of variables produced by the different methods. In the final model, we selected the clinical knowledge-driven approaches. Of the significant variables generated by the decision tree model, three variables were also selected by three models. In terms of model reliability, this apparent consistency between the different methods is relatively reassuring. A significant benefit of the approach driven by clinical knowledge is that it guarantees all variables are comprehensible to the clinician and practical within a standard hospital environment. Due to the limited number of events in our dataset, we chose not to divide the cohort into “development” and “validation” groups. This partitioning could potentially compromise the reliability of our regression analyses and is vulnerable to the fluctuations of random splitting. Therefore, for datasets with a limited number of events, we used bootstrapping to perform internal validation (Moons et al., 2012).

The Weng score incorporates a comprehensive set of risk factors, such as age, cancer, stroke, thrombophilia, HR > 100 bpm, paralysis, lower limb swelling, varicose veins, pleural effusion, trauma history, VTE history, strict bed rest >3 days, albumin <40 g/L, and D-dimer >0.5 μg/mL. The majority of the risk factors included in this analysis were also included in previous RAMs. Additionally, numerous risk factors were not included in the final model due to their absence from routine assessment upon admission in over 90% of individuals (contraceptive drug, hypertonic dehydration, unexplained habitual abortion, pregnancy or puerperium), cannot be quantified based on electronic health record (EHR) data, have poor inter-observer agreement (varicose veins, atrial fibrillation), or have low sample sizes (acute myocardial infarction, stroke, autoimmune disease, nephrotic syndrome, inflammatory bowel disease, sepsis, cardiac pacemaker). A number of “novel” risk factors present in the model could be linked to the severity of the disease or the risk of VTE in different populations (hypoalbuminemia (Lutsey & Zakai, 2023; Liu & Mi, 2021), elevated D-dimer (Francis et al., 2019; Cohen et al., 2014; Spyropoulos et al., 2020), pleural effusion). The incorporation of D-dimer into the IMPROVE VTE RAM, as proposed by Gibson et al. (2017) may enhance the capacity to categorise VTE risk in hospitalized patients.

Our findings confirm the limited predictive performance of the Caprini, Padua, Wells, Geneva and Autar scores in estimating the risk of VTE. This poor performance, particularly for the Caprini score in surgical patients, may be explained by differences in population. Caprini was developed primarily in elective surgical populations, whereas our surgical cohort included a mix of elective and emergency surgeries. In our study, in predicting VTE in medical patients, the Caprini score had the lowest sensitivity of 71% and Geneva score had the lowest specificity of 61.7%. In surgical patients, the Wells score had a sensitivity of only 28.4% and the Autar score had a specificity of 48.6%. The Weng score had a sensitivity and specificity of 80% or more, except for a slightly lower specificity in surgical patients. Sensitivity is critical in RAMs and can be used to select patients who can safely avoid prophylactic interventions (*i.e*., thromboprophylaxis). However, specificity should also be considered: using a score similar to the Autar score to determine thromboprophylaxis prescribing may lead to overtreatment due to its high sensitivity and low specificity. The current model contains some variables that are difficult to evaluate or very rare, which makes it difficult to score accurately in clinical use. The newly developed Weng score showed comparatively better performance than existing risk assessment models in this cohort by incorporating clinically relevant biomarkers (D-dimer, albumin) and novel risk factors (*e.g*., pleural effusion) that better reflect VTE pathophysiology, while overcoming the limitations of binary risk stratification seen in traditional models like Padua. While machine learning models show promise for VTE prediction, their “black box” nature and computational demands limit current clinical utility (Chen et al., 2024). In an informal internal time-motion assessment using retrospective chart data, we estimated that a clinician could calculate the Weng score in less than 2 min per patient using the article-based checklist. However, this was not a prospective implementation study; formal evaluation of clinical feasibility and workflow integration will require prospective testing.

The Caprini score, originally designed for surgical patients, has since been applied to medical patients as well. However, in our study, AUROC values for the Caprini score were only approximately 70% in medical patients and 65% in surgical patients. In addition, Caprini score has many evaluation indicators, and many variables are difficult to determine or specify, which brings challenges to the use it in clinical practice. The 2012 Guidelines for Antithrombotic Therapy and Thromboprophylaxis, published by the American College of Chest Physicians, indicate that the Padua score is an appropriate method for assessing VTE risk in medical inpatients (Kearon et al., 2012). The present study revealed that the Padua score exhibits superior performance to the Caprini score in medical patients. However, the Padua score showed better discrimination and sensitivity than the Caprini score in both medical and surgical patients, which may be attributed to the heterogeneity of the study populations. In addition, the Padua scale only categorises patients into high-risk and low-risk tiers, which may not provide clinicians with enough information to make more nuanced prevention decisions. Autar score is a widely used DVT RAM in medical or surgical patients. Based on the total score, patients are classified into different risk levels and preventive measures are taken accordingly. Autar is very similar to the score we developed and its predictive performance is close to Weng score in all five RAMs. This study shows that the Autar score has high sensitivity but low specificity, which may be a problem in clinical practice. PE is the most serious consequence of VTE, which is why clinicians are worried. Therefore, our study also further investigated the predictive role of Weng score and other five RAMs on PE. The Weng score maintains excellent performance in terms of discrimination, sensitivity, and specificity in predicting PE. The Wells and Geneva score are two commonly used clinical scoring systems to predict PE risk, but Coelho et al. (2020) have found that both scoring systems are less accurate (about 60%) in diagnosing PE in patients over 65 years of age, which is even lower than the accuracy found in our study. Girardi et al. (2020) also concluded that the Wells and Geneva scores are not reliable predictors of PE.

Model calibration is another important feature in assessing the clinical significance of predictive models. Well-calibrated models ensure that predicted risks are consistent with actual risks, which is particularly important for clinical decision-making as it affects the selection of therapeutic interventions and allocation of resources. In the calibration process, we first used the H-L test to assess the calibration of the six RAMs. The calibration curve provides a visual representation of the H-L test results and visually demonstrates the consistency of the model predicted probabilities with the actual observed values. Furthermore, a novel statistical method known as the GiViTI calibration band has been developed to overcome the limitations associated with the H-L test. In all three tests, Autar and Weng Score showed good calibration for VTE and PE, with Weng score favorable calibrated. Wells, Geneva and other RAMs are also reported to have poor calibration (Pandor et al., 2021). We stratified the Weng score and further confirmed that the predicted risk rankings corresponded well with the observed event rates across different risk categories within the sampled cohort. However, because the study used a case-control sampling design that oversampled VTE events, the numerical risk estimates (*e.g*., 85% in the very high-risk group) are not intended to represent absolute population risks. They serve primarily for relative risk stratification and ranking of patients. Similarly, the calibration curves and GiViTI calibration belts reflect goodness-of-fit for relative risk ordering within the sampled cohort; they do not guarantee absolute calibration in a general inpatient population. External validation in a cohort with natural event incidence is necessary to obtain clinically actionable absolute risk thresholds.

To date, few studies have investigated the net benefit of these RAMs in medical or surgical populations. Evaluating the net benefit of VTE diagnostic strategies will help determine the most clinically cost-effective approach (Goodacre et al., 2006). In two studies (Cheng et al., 2022; Wu & Cheng, 2023) of VTE prediction models in surgical disease, the authors’ VTE model showed a significantly higher net benefit than the Caprini score, whereas no comparative net benefit was reported for other RAMs. Our study is the first to comprehensively compare the net benefits of five conventional RAMs to Weng scores. It is critical to emphasize that all net benefit estimates are derived from a case-control sampled cohort in which VTE events were oversampled. Consequently, the predicted probabilities and net benefit curves reflect relative risk ordering among patients rather than absolute, actionable treatment thresholds. Within the limitations of the case-control sampling design, these results suggest that the Weng score may offer favorable relative clinical utility for risk stratification compared with previous RAMs. However, because the net benefit estimates depend on the predicted probabilities derived from a sampled cohort (with oversampled VTE events), the DCA findings should be interpreted as supporting relative risk ranking rather than establishing absolute treatment thresholds. External validation in a prospective representative cohort is required before the Weng score can be used to guide absolute clinical decision-making regarding thromboprophylaxis.

The current study presents several advantages. First, we not only introduce the RAM but also offer risk scoring and stratification. This enables sample-based risk estimation for relative risk stratification within the study cohort, facilitating the identification of individuals at higher relative risk who might be considered for pharmacological venous thrombosis prophylaxis. However, absolute predicted risks cannot be derived directly from this case-control sampled cohort; external calibration in a representative prospective cohort is needed to obtain clinically actionable absolute risk estimates. Second, the Weng score’s capacity to predict VTE risk on the first day of admission, irrespective of anticoagulation during the hospital stay, may help prioritize patients for further risk assessment. This finding was consistent with previous studies (Spyropoulos et al., 2011; Zakai et al., 2013), which have indicated that anticoagulation therapy has minimal impact on the performance of VTE models. The limited effect of anticoagulation on VTE model may be attributed to the fact that, while anticoagulation reduces VTE risk in randomized trials, the absolute magnitude of this change is relatively small (Laporte et al., 2011). Third, our study is a multi-center study. To make the predictors more convincing, we used three different variable selection methods, and consists of indicators that are easily accessible at admission. Fourth, we comprehensively compared Weng score performance with the five previous RAMs, and it had excellent performances for both VTE and PE in both medical and surgical patient populations. This study also has several limitations. First, as a retrospective case-control sampled cohort study, despite our adjustments for various potential confounders, the possibility of residual confounding remains. The 38% VTE proportion in the final sample reflects the sampling strategy and does not represent the true population incidence; accordingly, the absolute risk estimates derived from the model (*e.g*., risk stratification probabilities in Table 6) are conditional on the sampling design and should be interpreted as relative risk stratification rather than directly generalizable absolute risks. Calibration of absolute risks requires external validation in a prospective cohort with a representative event rate. Certain variable categorizations in our analysis (*e.g*., heart rate groupings based on the Wells scale, and trauma/surgery/bed rest categories based on the Padua scale) were adopted to maintain consistency with established clinical definitions and to facilitate direct comparison with existing RAMs. If this introduced any bias, it would likely favor the existing scores (Wells and Padua), as they were originally developed using these categorizations. Prospective studies are needed to confirm the findings and validate the model in real-time clinical settings. Second, the Weng score requires validation in broader populations. While our multicenter cohort included academic and regional hospitals, external validation in non-Chinese populations is essential to assess global applicability. We acknowledge that only internal validation was performed in this study, which limits the generalizability of our findings to other settings and populations. Therefore, we are planning a prospective multicenter external validation study in an independent cohort to further assess the model’s performance, including discrimination, calibration, and net benefit, across diverse clinical environments. We urge external validation in geographically diverse cohorts to ensure generalizability and mitigate overfitting risks. We acknowledge the critical importance of balancing thrombotic and bleeding risks-a limitation not addressed in our model, as the Weng score was developed specifically for VTE risk prediction and does not incorporate bleeding risk factors. The Weng score is not intended to replace clinical judgment regarding bleeding risk. Clinical decisions should integrate both considerations using validated bleeding risk tools (*e.g*., IMPROVE-BLEED, ABC-BLEED) alongside patient-specific factors. The Weng score is intended solely as a VTE risk stratification tool and a component of this comprehensive assessment; it is not a standalone decision aid for anticoagulation. Our model does not predict post-discharge VTE, future prospective validations will include 90-day follow-up. Furthermore, because DCA relies on calibrated probability estimates and our study used a case-control sampling design with oversampled VTE events, the net benefit curves presented in this study should be interpreted as reflecting relative clinical utility for risk stratification within the sampled cohort; they do not provide directly actionable absolute treatment thresholds. The absolute values of net benefit at specific threshold probabilities are conditional on the 38% VTE prevalence in our sample and would differ substantially in a general inpatient population with lower VTE incidence. External validation in a prospective cohort with representative event rates is necessary to assess DCA-based absolute net benefit and to determine clinically meaningful threshold probabilities for prophylaxis decisions.

In summary, we developed and internally validated a RAM for predicting VTE and PE, the Weng score. This model uses readily available risk factors to estimate the risk of VTE and PE in hospitalized patients from admission to discharge. This model had more favorable calibration and discrimination than Caprini, Padua, Wells, Geneva and Autar scores in our internal validation. Understanding the risk of VTE and PE is the first step to managing risk and improving health care safety. External validation across diverse populations and prospective studies incorporating anticoagulation management are essential.

## Supplemental Information

10.7717/peerj.21598/supp-1 Supplemental Information 1 Predictive variables for VTE were selected by LASSO binary regression analyses. (A) The curve of the coefficient path of 28 candidate variables in primary cohort. (B) The adjustment penalty parameter λ was selected in the LASSO model by 10-fold cross-validation.

10.7717/peerj.21598/supp-2 Supplemental Information 2 Calibration for VTE prediction by GiViTI calibration belt. A represents clinical knowledge-driven model; B represents data-driven model; C represents decision tree model.

10.7717/peerj.21598/supp-3 Supplemental Information 3 ROC curves for RAM prediction of VTE and PE in medical and surgical patients. A, B and C represent RAMs predicting VTE in all patients, internal medicine patients and surgical patients; D, E and F represent RAMs predicting PE in all patients, internal medicine patients and surgical patients. Under the case-control sampling design, the predicted probabilities reflect relative risk ranking; the net benefit curves should be interpreted as supporting relative clinical utility, and absolute threshold probabilities require external validation.

10.7717/peerj.21598/supp-4 Supplemental Information 4 Calibration plot for VTE prediction by Hosmer-Lemeshow test in all patients. The dashed diagonal line represents the ideal calibration where sample-based predicted probabilities perfectly match observed within sample outcomes. The green solid line depicts the model’s observed within sample performance, with closer alignment to the diagonal indicating better calibration. The predicted probabilities shown are derived from a case-control sampled cohort and reflect relative risk ordering; external calibration is needed to obtain absolute risk estimates.

10.7717/peerj.21598/supp-5 Supplemental Information 5 Calibration plot for VTE prediction by Hosmer-Lemeshow test in internal medicine patients. The dashed diagonal line represents the ideal calibration where sample-based predicted probabilities perfectly match observed within sample outcomes. The green solid line depicts the model’s observed within sample performance, with closer alignment to the diagonal indicating better calibration. The predicted probabilities shown are derived from a case-control sampled cohort and reflect relative risk ordering; external calibration is needed to obtain absolute risk estimates.

10.7717/peerj.21598/supp-6 Supplemental Information 6 Calibration plot for VTE prediction by Hosmer-Lemeshow test in surgical patients. The dashed diagonal line represents the ideal calibration where sample-based predicted probabilities perfectly match observed within sample outcomes. The green solid line depicts the model’s observed within sample performance, with closer alignment to the diagonal indicating better calibration. The predicted probabilities shown are derived from a case-control sampled cohort and reflect relative risk ordering; external calibration is needed to obtain absolute risk estimates.

10.7717/peerj.21598/supp-7 Supplemental Information 7 Calibration for VTE prediction by GiViTI calibration belt. A, B and C represent calibration of RAMs to predict VTE in all patients, internal medicine patients and surgical patients. The predicted probabilities shown are derived from a case-control sampled cohort and reflect relative risk ordering; external calibration is needed to obtain absolute risk estimates.

10.7717/peerj.21598/supp-8 Supplemental Information 8 Calibration plot for PE prediction by Hosmer-Lemeshow test in all patients. The dashed diagonal line represents the ideal calibration where sample-based predicted probabilities perfectly match observed within sample outcomes. The green solid line depicts the model’s observed within sample performance, with closer alignment to the diagonal indicating better calibration. The predicted probabilities shown are derived from a case-control sampled cohort and reflect relative risk ordering; external calibration is needed to obtain absolute risk estimates.

10.7717/peerj.21598/supp-9 Supplemental Information 9 Calibration plot for PE prediction by Hosmer-Lemeshow test in internal medicine patients. The dashed diagonal line represents the ideal calibration where sample-based predicted probabilities perfectly match observed within sample outcomes. The green solid line depicts the model’s observed within sample performance, with closer alignment to the diagonal indicating better calibration. The predicted probabilities shown are derived from a case-control sampled cohort and reflect relative risk ordering; external calibration is needed to obtain absolute risk estimates.

10.7717/peerj.21598/supp-10 Supplemental Information 10 Calibration plot for PE prediction by Hosmer-Lemeshow test in surgical patients. The dashed diagonal line represents the ideal calibration where sample-based predicted probabilities perfectly match observed within sample outcomes. The green solid line depicts the model’s observed within sample performance, with closer alignment to the diagonal indicating better calibration. The predicted probabilities shown are derived from a case-control sampled cohort and reflect relative risk ordering; external calibration is needed to obtain absolute risk estimates.

10.7717/peerj.21598/supp-11 Supplemental Information 11 Calibration for PE prediction by GiViTI calibration belt. A, B and C represent calibration of RAMs to predict PE in all patients, internal medicine patients and surgical patients. The predicted probabilities shown are derived from a case-control sampled cohort and reflect relative risk ordering; external calibration is needed to obtain absolute risk estimates.

10.7717/peerj.21598/supp-12 Supplemental Information 12 Demographic and clinical characteristics of the patients with thromboembolism event.

10.7717/peerj.21598/supp-13 Supplemental Information 13 Multivariate regression analysis for thromboembolism events (Data-driven).

10.7717/peerj.21598/supp-14 Supplemental Information 14 Performance comparison of six scores for predicting thromboembolism (*n* = 1,791).

10.7717/peerj.21598/supp-15 Supplemental Information 15 Performance comparison of six scores for predicting thromboembolism in internal medicine population (*n* = 1,070).

10.7717/peerj.21598/supp-16 Supplemental Information 16 Performance comparison of six scores for predicting thromboembolism in surgical population (*n* = 721).

10.7717/peerj.21598/supp-17 Supplemental Information 17 Performance comparison of six scores for predicting PE (*n* = 1,274).

10.7717/peerj.21598/supp-18 Supplemental Information 18 Performance comparison of six scores for predicting PE in internal medicine population (*n* = 842).

10.7717/peerj.21598/supp-19 Supplemental Information 19 Performance comparison of six scores for predicting PE in surgical population (*n* = 432).

10.7717/peerj.21598/supp-20 Supplemental Information 20 Raw data. 血栓数据更新 = Thrombosis Data Update

10.7717/peerj.21598/supp-21 Supplemental Information 21 Codebook. This codebook describes every variable in the raw dataset.

10.7717/peerj.21598/supp-22 Supplemental Information 22 Clear explanation of the analytic population for PE prediction.

10.7717/peerj.21598/supp-23 Supplemental Information 23 Corrected and reproducible Weng score derivation.

## Additional Information and Declarations

### Data Availability

The following information was supplied regarding data availability:

The data is available in the Supplemental Files.
