# Supplementary material for: Derivation of a novel risk assessment model of venous thromboembolism in hospitalized patients
Source: PeerJ. 2026 Aug 4;14:e21598. doi: 10.7717/peerj.21598 (PMC13450040; doi:10.7717/peerj.21598)
Supplement: Supplementary file 1 [file peerj-14-21598-s001.pdf]

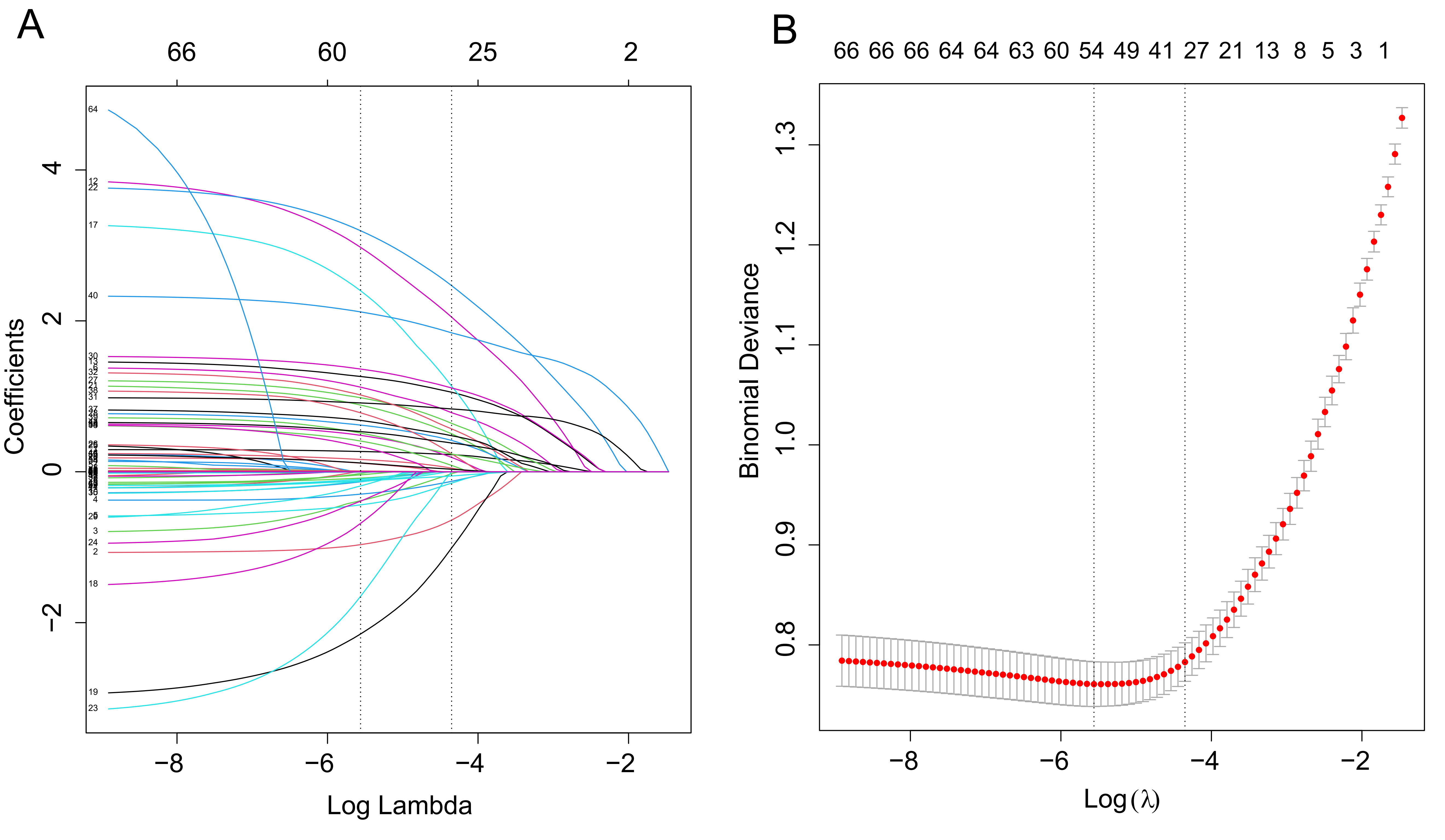
A
66 60 25 2
Coefficients
Log Lambda
−8 −6 −4 −2
4 2 0 −2
B
66 66 66 64 64 63 60 54 49 41 27 21 13 8 5 3 1
Binomial Deviance
Log(λ)
1.3 1.2 1.1 1.0 0.9 0.8
