# Supplementary material for: Derivation of a novel risk assessment model of venous thromboembolism in hospitalized patients
Source: PeerJ. 2026 Aug 4;14:e21598. doi: 10.7717/peerj.21598 (PMC13450040; doi:10.7717/peerj.21598)
Supplement: Supplementary file 2 [file peerj-14-21598-s002.pdf]

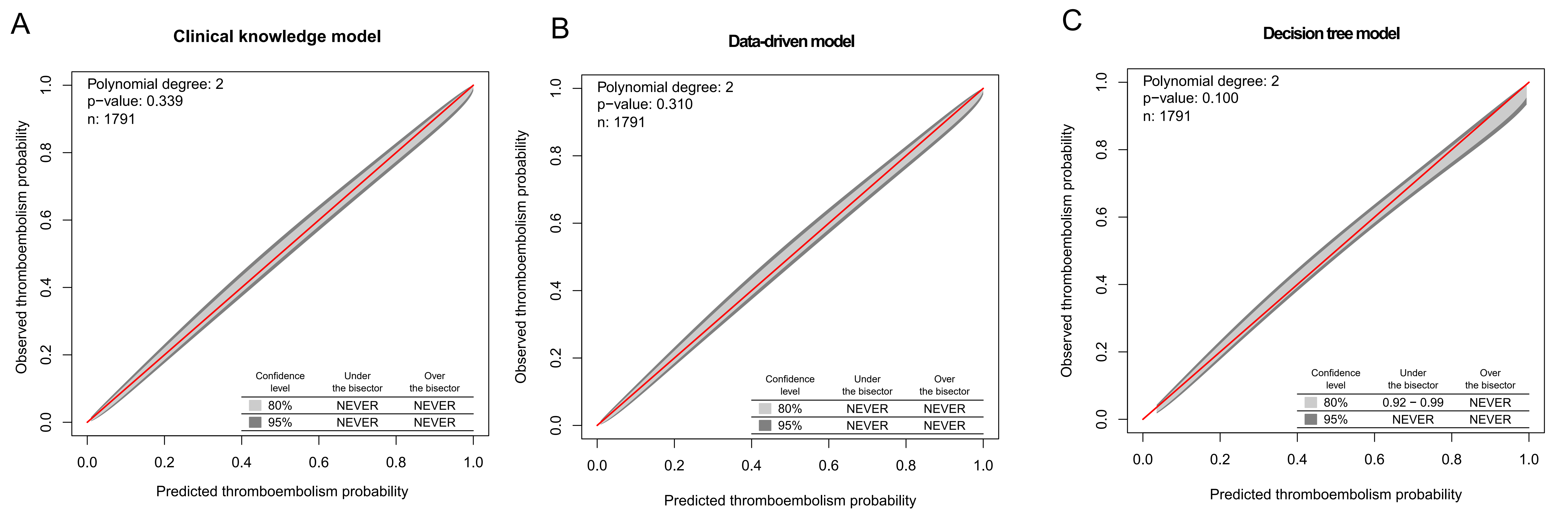
A
Clinical knowledge model
Polynomial degree: 2
p−value: 0.339
n: 1791
Observed thromboembolism probability
Predicted thromboembolism probability
0.0
0.2
0.4
0.6
0.8
1.0
Confidence level
Under the bisector
Over the bisector
80% NEVER NEVER
95% NEVER NEVER
B
Data-driven model
Polynomial degree: 2
p−value: 0.310
n: 1791
Observed thromboembolism probability
Predicted thromboembolism probability
Confidence level
Under the bisector
Over the bisector
80% NEVER NEVER
95% NEVER NEVER
C
Decision tree model
Polynomial degree: 2
p−value: 0.100
n: 1791
Observed thromboembolism probability
Predicted thromboembolism probability
Confidence level
Under the bisector
Over the bisector
80% 0.92 − 0.99 NEVER
95% NEVER NEVER
