# Supplementary material for: Derivation of a novel risk assessment model of venous thromboembolism in hospitalized patients
Source: PeerJ. 2026 Aug 4;14:e21598. doi: 10.7717/peerj.21598 (PMC13450040; doi:10.7717/peerj.21598)
Supplement: Supplementary file 3 [file peerj-14-21598-s003.pdf]

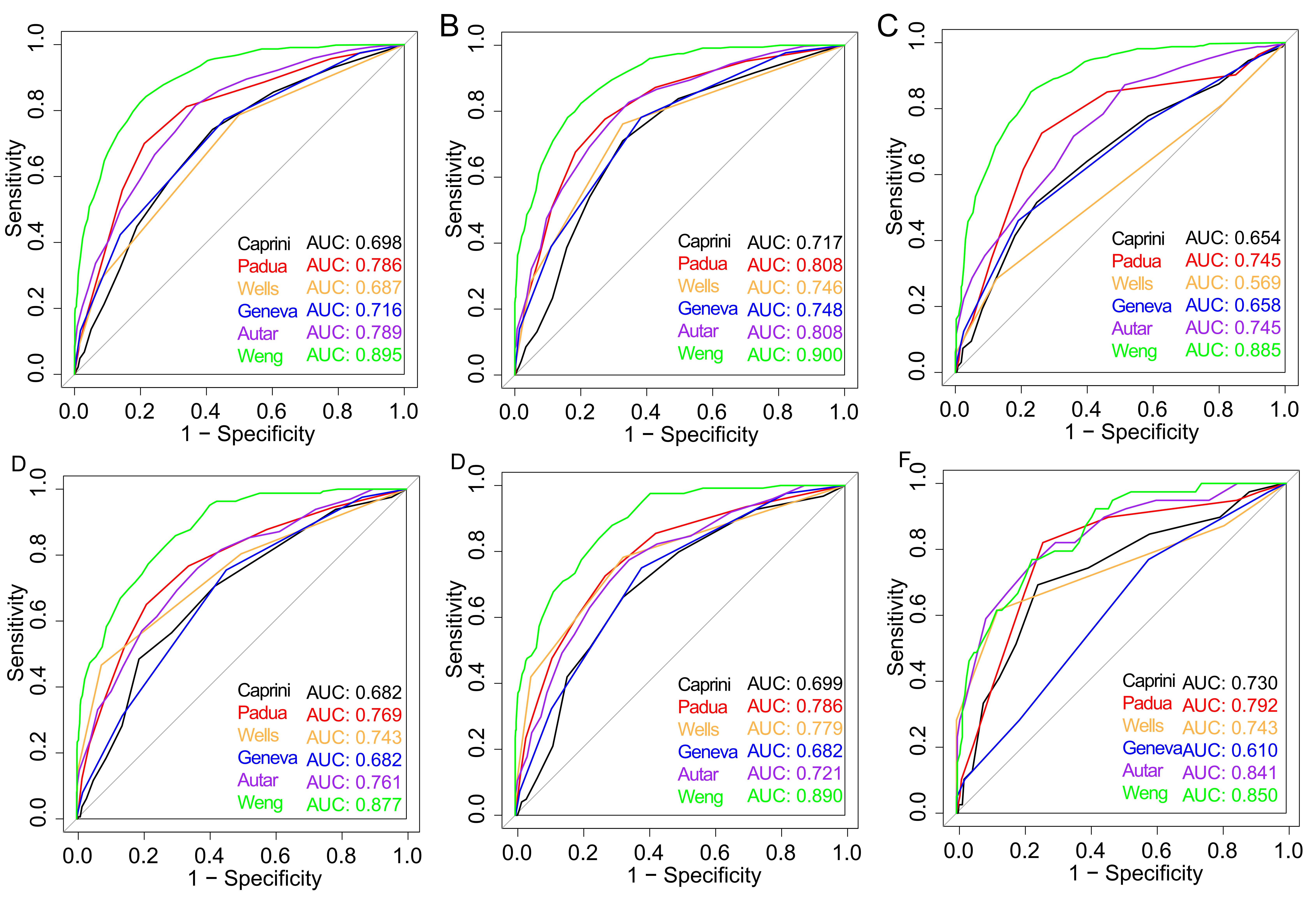
B
C
D
D
F
Sensitivity
1 − Specificity
0.0
0.2
0.4
0.6
0.8
1.0
Caprini AUC: 0.698
Padua AUC: 0.786
Wells AUC: 0.687
Geneva AUC: 0.716
Autar AUC: 0.789
Weng AUC: 0.895
Caprini AUC: 0.717
Padua AUC: 0.808
Wells AUC: 0.746
Geneva AUC: 0.748
Autar AUC: 0.808
Weng AUC: 0.900
Caprini AUC: 0.654
Padua AUC: 0.745
Wells AUC: 0.569
Geneva AUC: 0.658
Autar AUC: 0.745
Weng AUC: 0.885
Caprini AUC: 0.682
Padua AUC: 0.769
Wells AUC: 0.743
Geneva AUC: 0.682
Autar AUC: 0.761
Weng AUC: 0.877
Caprini AUC: 0.699
Padua AUC: 0.786
Wells AUC: 0.779
Geneva AUC: 0.682
Autar AUC: 0.721
Weng AUC: 0.890
Caprini AUC: 0.730
Padua AUC: 0.792
Wells AUC: 0.743
Geneva AUC: 0.610
Autar AUC: 0.841
Weng AUC: 0.850
