# Supplementary material for: Derivation of a novel risk assessment model of venous thromboembolism in hospitalized patients
Source: PeerJ. 2026 Aug 4;14:e21598. doi: 10.7717/peerj.21598 (PMC13450040; doi:10.7717/peerj.21598)
Supplement: Supplementary file 5 [file peerj-14-21598-s005.pdf]

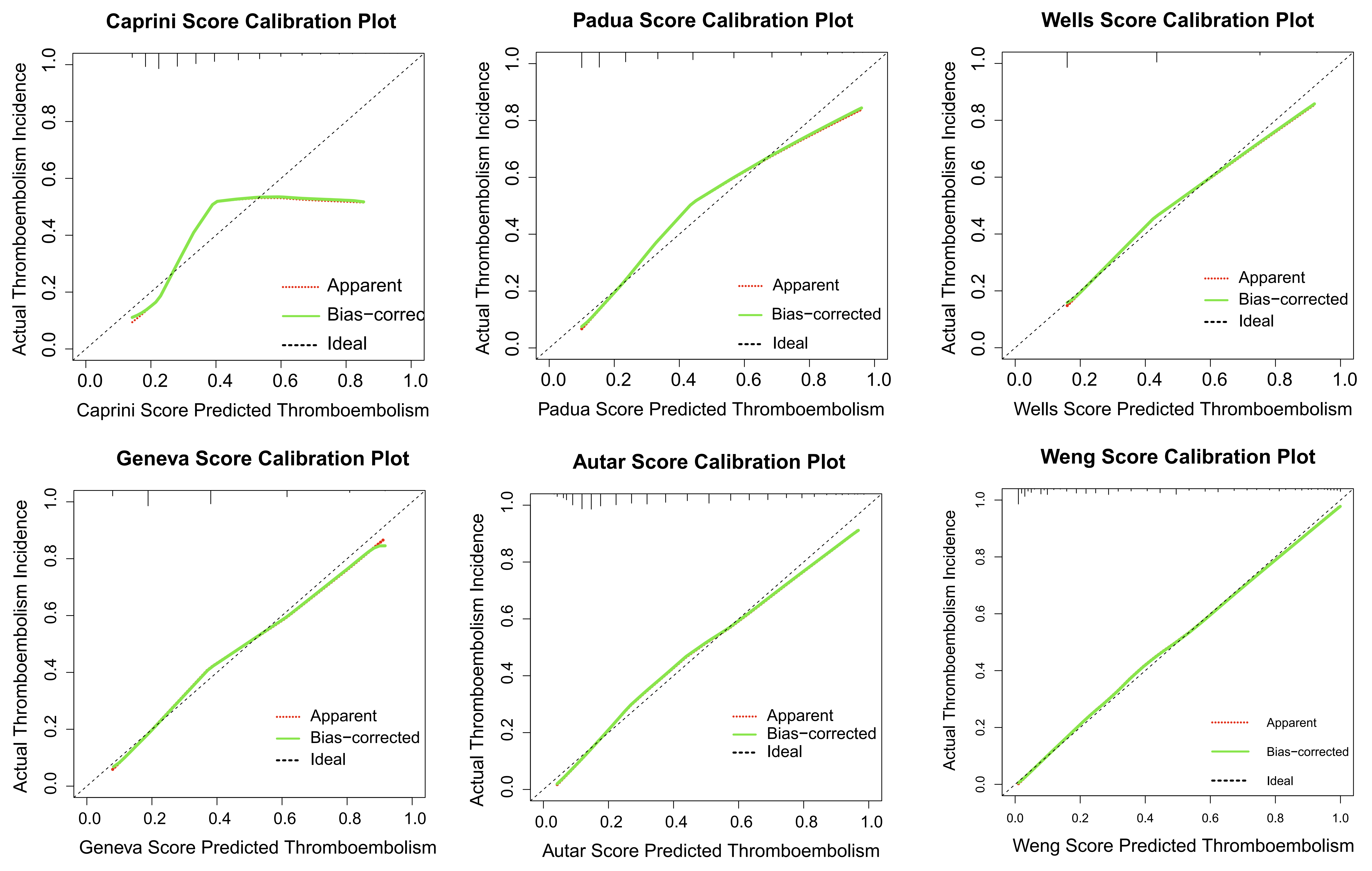

Caprini Score Calibration Plot
Actual Thromboembolism Incidence
0.0
0.2
0.4
0.6
0.8
1.0
Apparent
Bias−correc
Ideal
Caprini Score Predicted Thromboembolism
Padua Score Calibration Plot
Actual Thromboembolism Incidence
Apparent
Bias−corrected
Ideal
Padua Score Predicted Thromboembolism
Wells Score Calibration Plot
Actual Thromboembolism Incidence
Apparent
Bias−corrected
Ideal
Wells Score Predicted Thromboembolism
Geneva Score Calibration Plot
Actual Thromboembolism Incidence
Apparent
Bias−corrected
Ideal
Geneva Score Predicted Thromboembolism
Autar Score Calibration Plot
Actual Thromboembolism Incidence
Apparent
Bias−corrected
Ideal
Autar Score Predicted Thromboembolism
Weng Score Calibration Plot
Actual Thromboembolism Incidence
Apparent
Bias−corrected
Ideal
Weng Score Predicted Thromboembolism
