# Supplementary material for: Derivation of a novel risk assessment model of venous thromboembolism in hospitalized patients
Source: PeerJ. 2026 Aug 4;14:e21598. doi: 10.7717/peerj.21598 (PMC13450040; doi:10.7717/peerj.21598)
Supplement: Supplementary file 6 [file peerj-14-21598-s006.pdf]

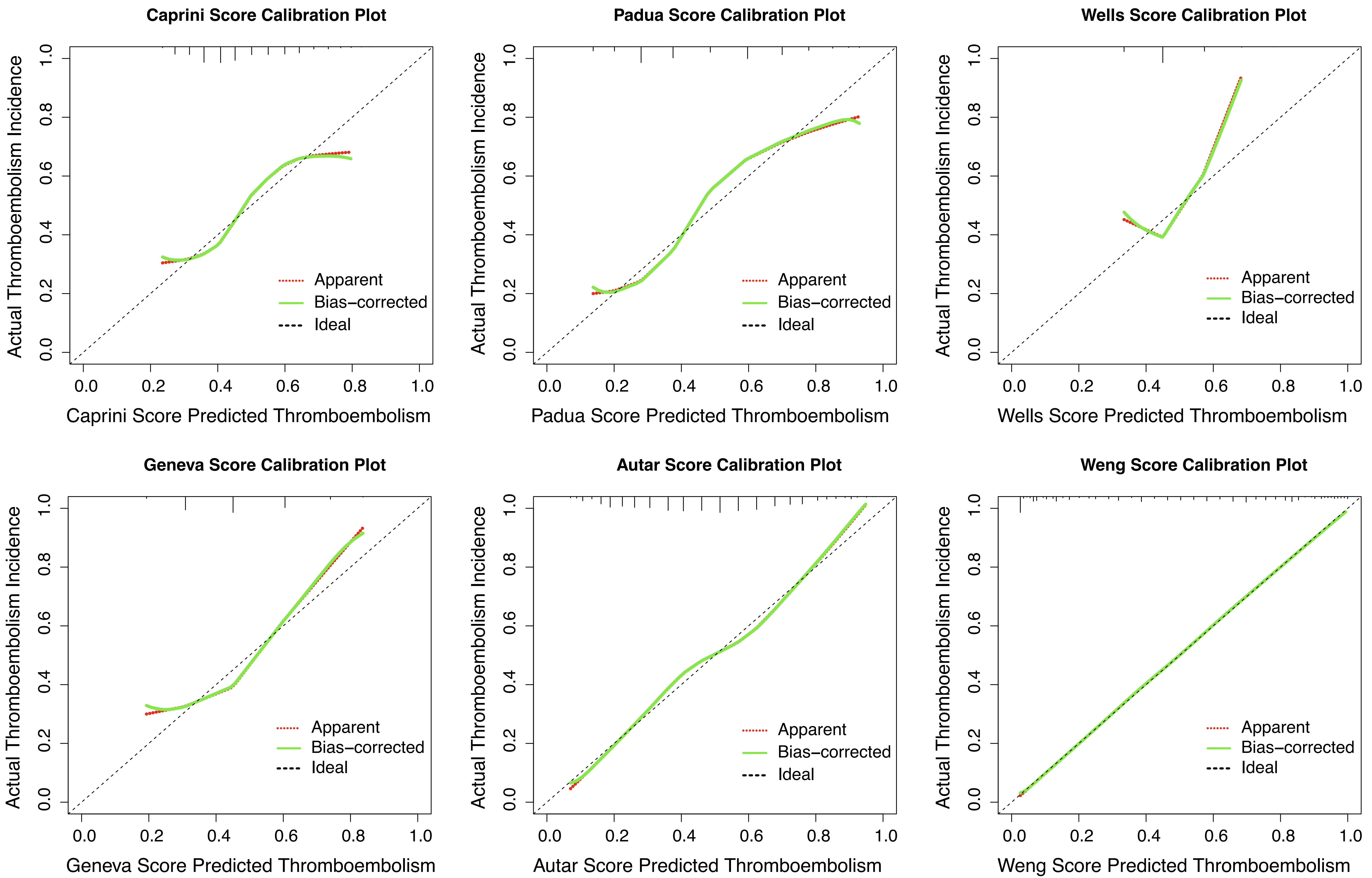

Caprini Score Calibration Plot
Actual Thromboembolism Incidence
Caprini Score Predicted Thromboembolism
Padua Score Calibration Plot
Padua Score Predicted Thromboembolism
Wells Score Calibration Plot
Wells Score Predicted Thromboembolism
Geneva Score Calibration Plot
Geneva Score Predicted Thromboembolism
Autar Score Calibration Plot
Autar Score Predicted Thromboembolism
Weng Score Calibration Plot
Weng Score Predicted Thromboembolism
Apparent
Bias−corrected
Ideal
0.0
0.2
0.4
0.6
0.8
1.0
