# Supplementary material for: Derivation of a novel risk assessment model of venous thromboembolism in hospitalized patients
Source: PeerJ. 2026 Aug 4;14:e21598. doi: 10.7717/peerj.21598 (PMC13450040; doi:10.7717/peerj.21598)
Supplement: Supplementary file 7 [file peerj-14-21598-s007.pdf]

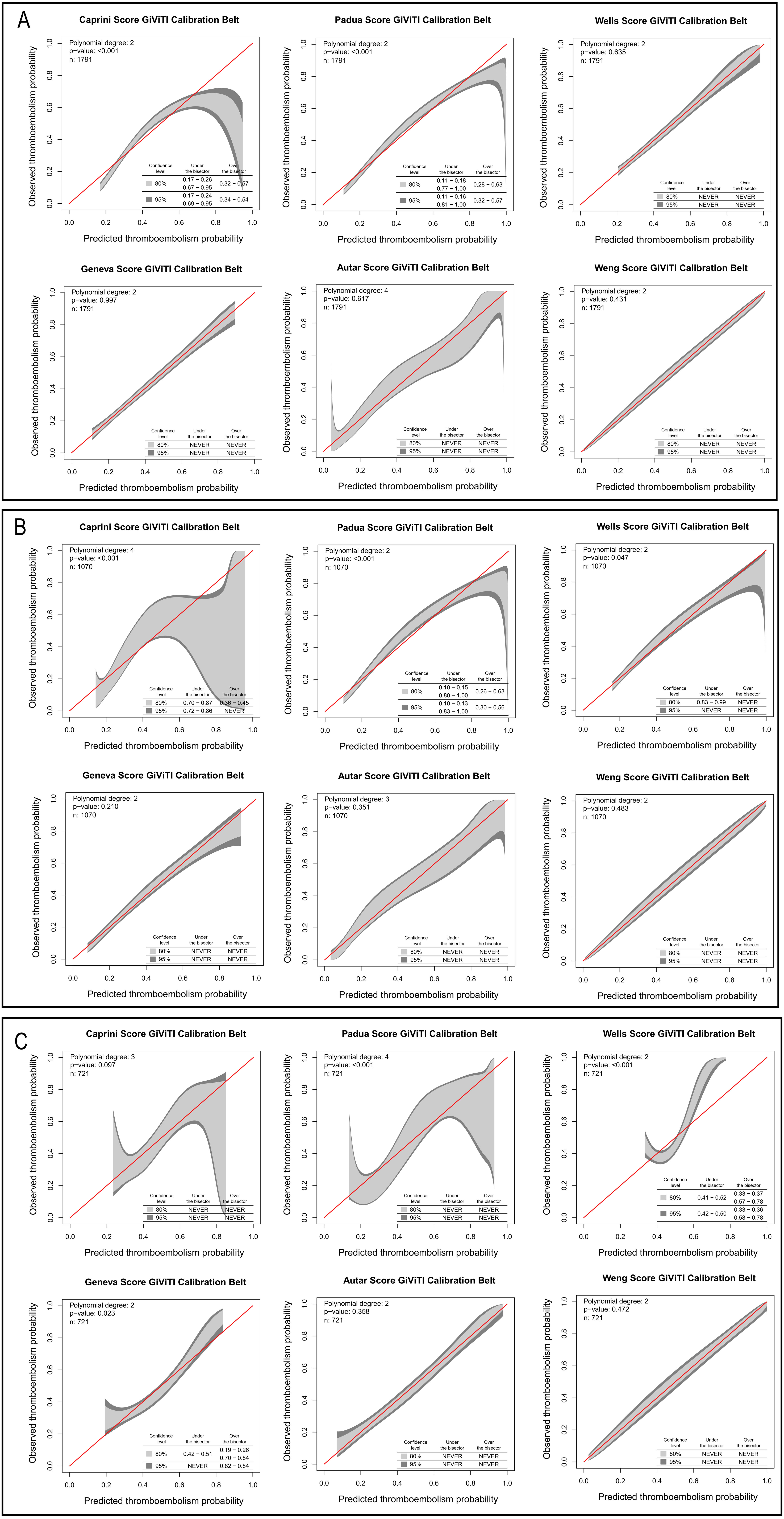
A
Caprini Score GiViTI Calibration Belt
Polynomial degree: 2
p-value: <0.001
n: 1791
Confidence level | Under the bisector | Over the bisector
80% | 0.17 – 0.26, 0.67 – 0.95 | 0.32 – 0.57
95% | 0.17 – 0.24, 0.69 – 0.95 | 0.34 – 0.54
Padua Score GiViTI Calibration Belt
Polynomial degree: 2
p-value: <0.001
n: 1791
80% | 0.11 – 0.18, 0.77 – 1.00 | 0.28 – 0.63
95% | 0.11 – 0.16, 0.81 – 1.00 | 0.32 – 0.57
Wells Score GiViTI Calibration Belt
Polynomial degree: 2
p-value: 0.635
n: 1791
80% | NEVER | NEVER
95% | NEVER | NEVER
Geneva Score GiViTI Calibration Belt
Polynomial degree: 2
p-value: 0.997
n: 1791
80% | NEVER | NEVER
95% | NEVER | NEVER
Autar Score GiViTI Calibration Belt
Polynomial degree: 4
p-value: 0.617
n: 1791
80% | NEVER | NEVER
95% | NEVER | NEVER
Weng Score GiViTI Calibration Belt
Polynomial degree: 2
p-value: 0.431
n: 1791
80% | NEVER | NEVER
95% | NEVER | NEVER
B
Caprini Score GiViTI Calibration Belt
Polynomial degree: 4
p-value: <0.001
n: 1070
80% | 0.70 – 0.87 | 0.36 – 0.45
95% | 0.72 – 0.86 | NEVER
Padua Score GiViTI Calibration Belt
Polynomial degree: 2
p-value: <0.001
n: 1070
80% | 0.10 – 0.15, 0.80 – 1.00 | 0.26 – 0.63
95% | 0.10 – 0.13, 0.83 – 1.00 | 0.30 – 0.56
Wells Score GiViTI Calibration Belt
Polynomial degree: 2
p-value: 0.047
n: 1070
80% | 0.83 – 0.99 | NEVER
95% | NEVER | NEVER
Geneva Score GiViTI Calibration Belt
Polynomial degree: 2
p-value: 0.210
n: 1070
80% | NEVER | NEVER
95% | NEVER | NEVER
Autar Score GiViTI Calibration Belt
Polynomial degree: 3
p-value: 0.351
n: 1070
80% | NEVER | NEVER
95% | NEVER | NEVER
Weng Score GiViTI Calibration Belt
Polynomial degree: 2
p-value: 0.483
n: 1070
80% | NEVER | NEVER
95% | NEVER | NEVER
C
Caprini Score GiViTI Calibration Belt
Polynomial degree: 3
p-value: 0.097
n: 721
80% | NEVER | NEVER
95% | NEVER | NEVER
Padua Score GiViTI Calibration Belt
Polynomial degree: 4
p-value: <0.001
n: 721
80% | NEVER | NEVER
95% | NEVER | NEVER
Wells Score GiViTI Calibration Belt
Polynomial degree: 2
p-value: <0.001
n: 721
80% | 0.41 – 0.52 | 0.33 – 0.37, 0.57 – 0.78
95% | 0.42 – 0.50 | 0.33 – 0.36, 0.58 – 0.78
Geneva Score GiViTI Calibration Belt
Polynomial degree: 2
p-value: 0.023
n: 721
80% | 0.42 – 0.51 | 0.19 – 0.26, 0.70 – 0.84
95% | NEVER | 0.82 – 0.84
Autar Score GiViTI Calibration Belt
Polynomial degree: 2
p-value: 0.358
n: 721
80% | NEVER | NEVER
95% | NEVER | NEVER
Weng Score GiViTI Calibration Belt
Polynomial degree: 2
p-value: 0.472
n: 721
80% | NEVER | NEVER
95% | NEVER | NEVER
Observed thromboembolism probability
Predicted thromboembolism probability
0.0
0.2
0.4
0.6
0.8
1.0
