# Supplementary material for: Derivation of a novel risk assessment model of venous thromboembolism in hospitalized patients
Source: PeerJ. 2026 Aug 4;14:e21598. doi: 10.7717/peerj.21598 (PMC13450040; doi:10.7717/peerj.21598)
Supplement: Supplementary file 9 [file peerj-14-21598-s009.pdf]

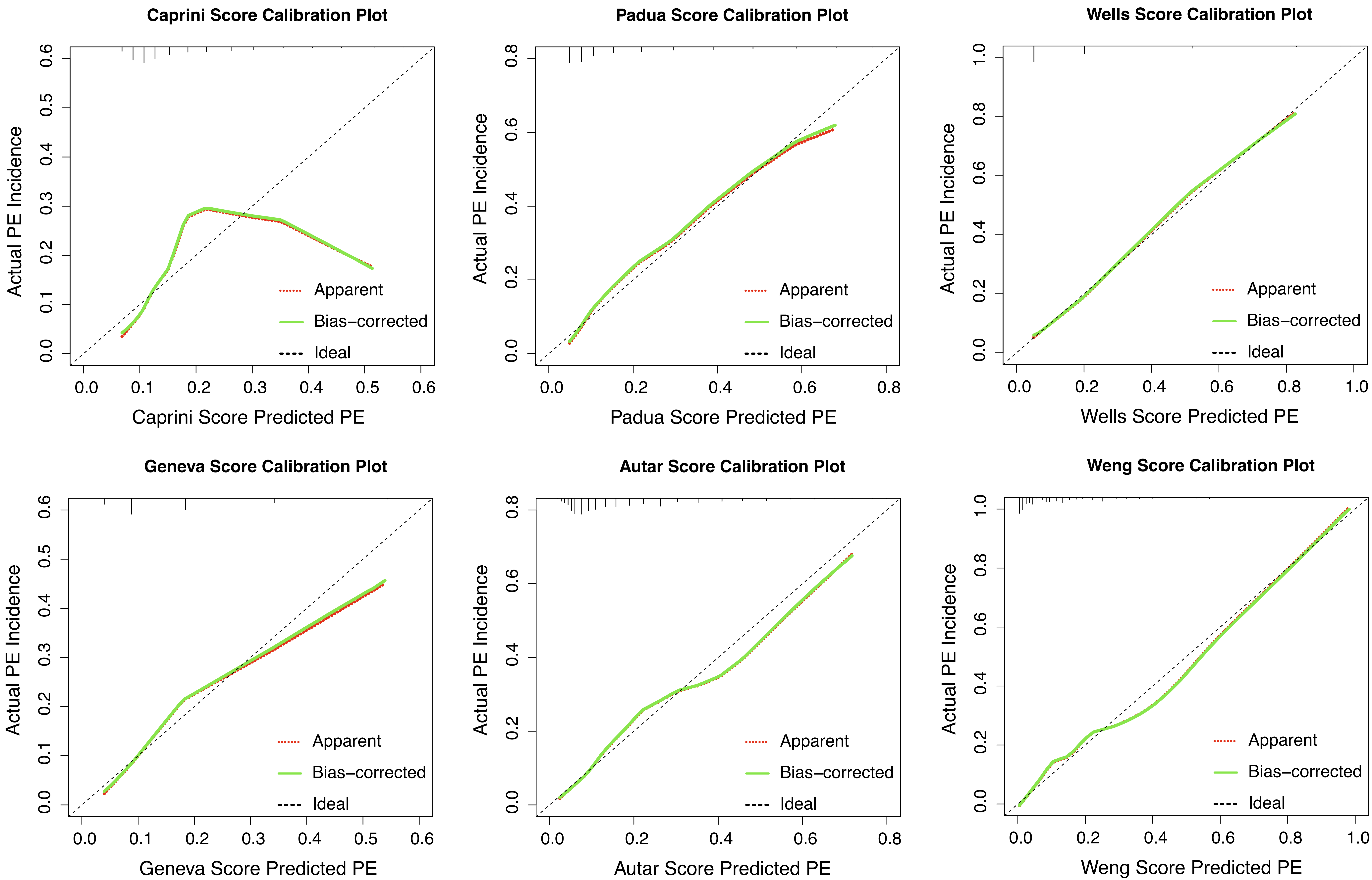
Caprini Score Calibration Plot
Actual PE Incidence
Caprini Score Predicted PE
Padua Score Calibration Plot
Padua Score Predicted PE
Wells Score Calibration Plot
Wells Score Predicted PE
Geneva Score Calibration Plot
Geneva Score Predicted PE
Autar Score Calibration Plot
Autar Score Predicted PE
Weng Score Calibration Plot
Weng Score Predicted PE
Apparent
Bias−corrected
Ideal
