# Supplementary material for: Derivation of a novel risk assessment model of venous thromboembolism in hospitalized patients
Source: PeerJ. 2026 Aug 4;14:e21598. doi: 10.7717/peerj.21598 (PMC13450040; doi:10.7717/peerj.21598)
Supplement: Supplementary file 10 [file peerj-14-21598-s010.pdf]

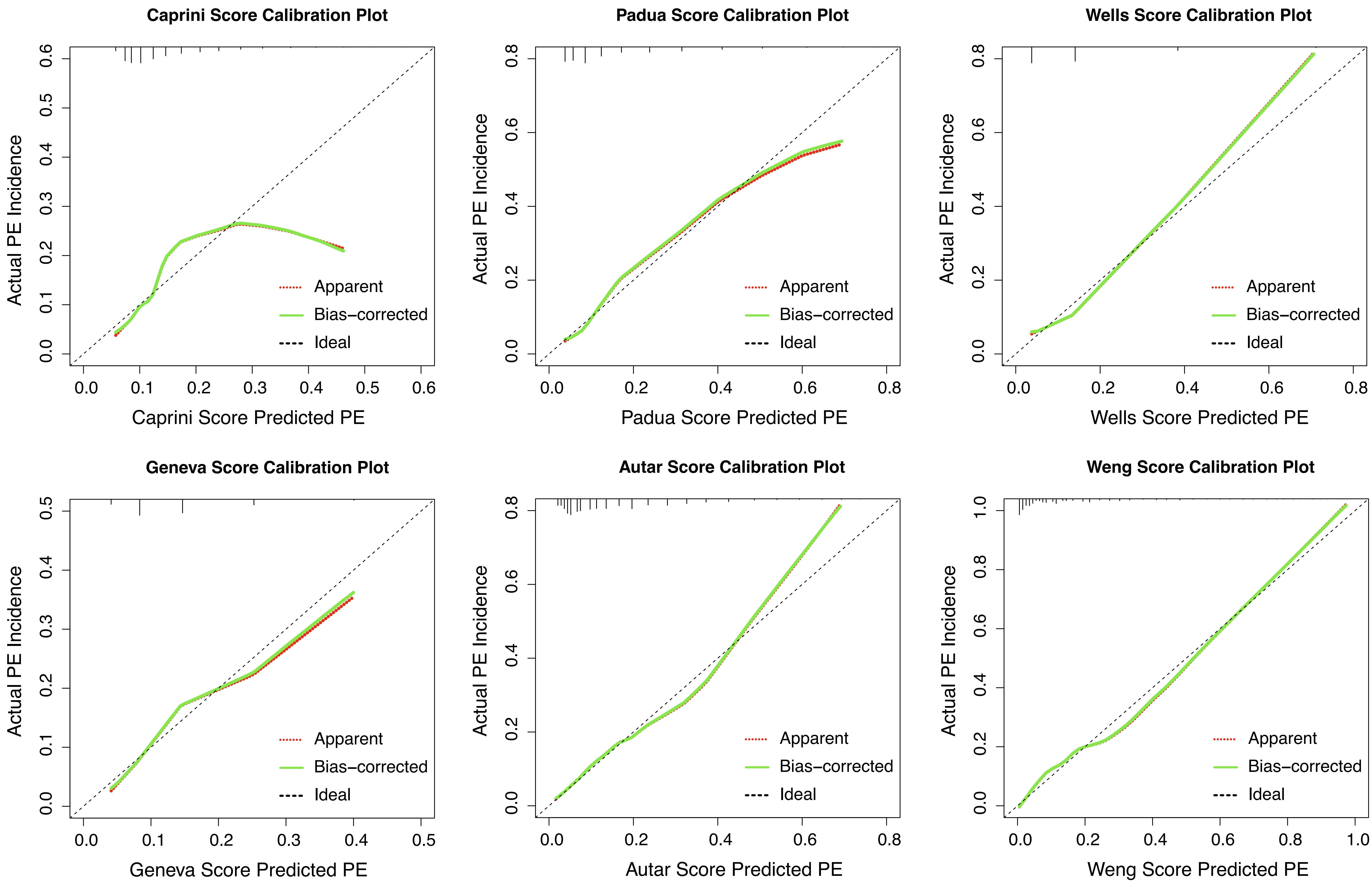
Caprini Score Calibration Plot
Actual PE Incidence
Caprini Score Predicted PE
0.0
0.1
0.2
0.3
0.4
0.5
0.6
Apparent
Bias−corrected
Ideal
Padua Score Calibration Plot
Actual PE Incidence
Padua Score Predicted PE
0.0
0.2
0.4
0.6
0.8
Apparent
Bias−corrected
Ideal
Wells Score Calibration Plot
Actual PE Incidence
Wells Score Predicted PE
0.0
0.2
0.4
0.6
0.8
Apparent
Bias−corrected
Ideal
Geneva Score Calibration Plot
Actual PE Incidence
Geneva Score Predicted PE
0.0
0.1
0.2
0.3
0.4
0.5
Apparent
Bias−corrected
Ideal
Autar Score Calibration Plot
Actual PE Incidence
Autar Score Predicted PE
0.0
0.2
0.4
0.6
0.8
Apparent
Bias−corrected
Ideal
Weng Score Calibration Plot
Actual PE Incidence
Weng Score Predicted PE
0.0
0.2
0.4
0.6
0.8
1.0
Apparent
Bias−corrected
Ideal
