# Supplementary material for: Derivation of a novel risk assessment model of venous thromboembolism in hospitalized patients
Source: PeerJ. 2026 Aug 4;14:e21598. doi: 10.7717/peerj.21598 (PMC13450040; doi:10.7717/peerj.21598)
Supplement: Supplementary file 11 [file peerj-14-21598-s011.pdf]

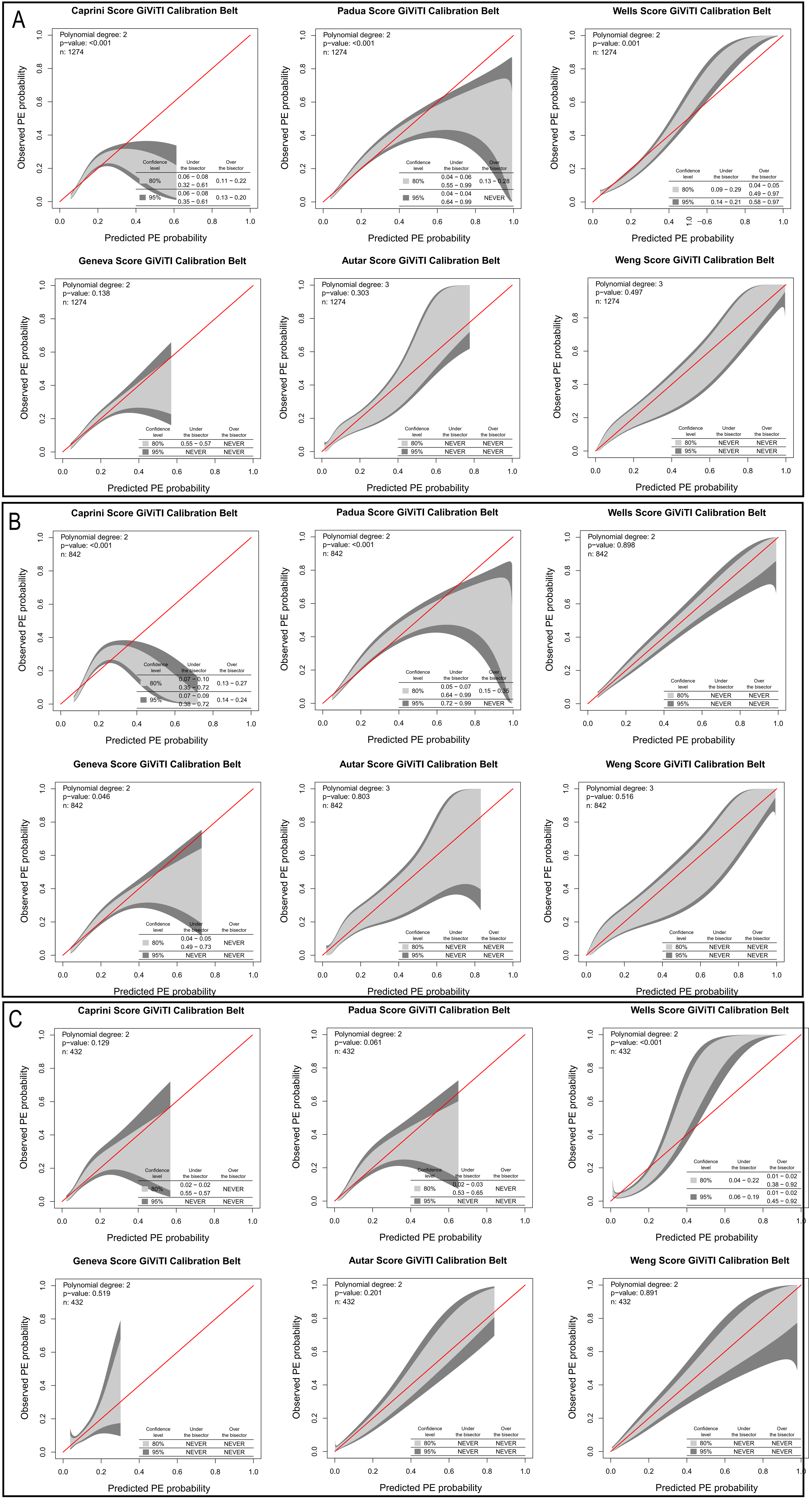
A
Caprini Score GiViTI Calibration Belt
Polynomial degree: 2
p-value: <0.001
n: 1274
Confidence level | Under the bisector | Over the bisector
80% | 0.06 – 0.08 | 0.11 – 0.22
0.32 – 0.61
95% | 0.06 – 0.08 | 0.13 – 0.20
0.35 – 0.61
Padua Score GiViTI Calibration Belt
Polynomial degree: 2
p-value: <0.001
n: 1274
Confidence level | Under the bisector | Over the bisector
80% | 0.04 – 0.06 | 0.13 – 0.28
0.55 – 0.99
95% | 0.04 – 0.04 | NEVER
0.64 – 0.99
Wells Score GiViTI Calibration Belt
Polynomial degree: 2
p-value: 0.001
n: 1274
Confidence level | Under the bisector | Over the bisector
80% | 0.09 – 0.29 | 0.04 – 0.05
0.49 – 0.97
95% | 0.14 – 0.21 | 0.58 – 0.97
Geneva Score GiViTI Calibration Belt
Polynomial degree: 2
p-value: 0.138
n: 1274
Confidence level | Under the bisector | Over the bisector
80% | 0.55 – 0.57 | NEVER
95% | NEVER | NEVER
Autar Score GiViTI Calibration Belt
Polynomial degree: 3
p-value: 0.303
n: 1274
Confidence level | Under the bisector | Over the bisector
80% | NEVER | NEVER
95% | NEVER | NEVER
Weng Score GiViTI Calibration Belt
Polynomial degree: 3
p-value: 0.497
n: 1274
Confidence level | Under the bisector | Over the bisector
80% | NEVER | NEVER
95% | NEVER | NEVER
B
Caprini Score GiViTI Calibration Belt
Polynomial degree: 2
p-value: <0.001
n: 842
Confidence level | Under the bisector | Over the bisector
80% | 0.07 – 0.10 | 0.13 – 0.27
0.35 – 0.72
95% | 0.07 – 0.09 | 0.14 – 0.24
0.38 – 0.72
Padua Score GiViTI Calibration Belt
Polynomial degree: 2
p-value: <0.001
n: 842
Confidence level | Under the bisector | Over the bisector
80% | 0.05 – 0.07 | 0.15 – 0.35
0.64 – 0.99
95% | 0.72 – 0.99 | NEVER
Wells Score GiViTI Calibration Belt
Polynomial degree: 2
p-value: 0.898
n: 842
Confidence level | Under the bisector | Over the bisector
80% | NEVER | NEVER
95% | NEVER | NEVER
Geneva Score GiViTI Calibration Belt
Polynomial degree: 2
p-value: 0.046
n: 842
Confidence level | Under the bisector | Over the bisector
80% | 0.04 – 0.05 | NEVER
0.49 – 0.73
95% | NEVER | NEVER
Autar Score GiViTI Calibration Belt
Polynomial degree: 3
p-value: 0.803
n: 842
Confidence level | Under the bisector | Over the bisector
80% | NEVER | NEVER
95% | NEVER | NEVER
Weng Score GiViTI Calibration Belt
Polynomial degree: 3
p-value: 0.516
n: 842
Confidence level | Under the bisector | Over the bisector
80% | NEVER | NEVER
95% | NEVER | NEVER
C
Caprini Score GiViTI Calibration Belt
Polynomial degree: 2
p-value: 0.129
n: 432
Confidence level | Under the bisector | Over the bisector
80% | 0.02 – 0.02 | NEVER
0.55 – 0.57
95% | NEVER | NEVER
Padua Score GiViTI Calibration Belt
Polynomial degree: 2
p-value: 0.061
n: 432
Confidence level | Under the bisector | Over the bisector
80% | 0.02 – 0.03 | NEVER
0.53 – 0.65
95% | NEVER | NEVER
Wells Score GiViTI Calibration Belt
Polynomial degree: 2
p-value: <0.001
n: 432
Confidence level | Under the bisector | Over the bisector
80% | 0.04 – 0.22 | 0.01 – 0.02
0.38 – 0.92
95% | 0.06 – 0.19 | 0.01 – 0.02
0.45 – 0.92
Geneva Score GiViTI Calibration Belt
Polynomial degree: 2
p-value: 0.519
n: 432
Confidence level | Under the bisector | Over the bisector
80% | NEVER | NEVER
95% | NEVER | NEVER
Autar Score GiViTI Calibration Belt
Polynomial degree: 2
p-value: 0.201
n: 432
Confidence level | Under the bisector | Over the bisector
80% | NEVER | NEVER
95% | NEVER | NEVER
Weng Score GiViTI Calibration Belt
Polynomial degree: 2
p-value: 0.891
n: 432
Confidence level | Under the bisector | Over the bisector
80% | NEVER | NEVER
95% | NEVER | NEVER
Observed PE probability
Predicted PE probability
0.0
0.2
0.4
0.6
0.8
1.0
