# Supplementary material for: Derivation of a novel risk assessment model of venous thromboembolism in hospitalized patients
Source: PeerJ. 2026 Aug 4;14:e21598. doi: 10.7717/peerj.21598 (PMC13450040; doi:10.7717/peerj.21598)
Supplement: Supplementary file 12 [file peerj-14-21598-s012.docx]

Table S1 Demographic and clinical characteristics of the patients with thromboembolism event

| Variables | Non VTE (n=1111) | VTE (n=680) | *P* value |
| --- | --- | --- | --- |
| Demographics |  |  |  |
| Age (years), Median (IQR) | 60 (48, 70) | 70 (59, 79) | <0.001 |
| Male, n (%) | 623 (56.1) | 375 (55.1) | 0.701 |
| BMI (kg/m2), Median (IQR) | 23.51 (21.22, 26.04) | 23.34 (20.83, 25.59) | 0.039 |
| Smoking, n (%) | 217 (19.5) | 121 (17.8) | 0.362 |
| Drinking, n (%) | 188 (16.9) | 98 (14.4) | 0.159 |
| Chronic / acute comorbidities |  |  |  |
| Diabetes mellitus, n (%) | 324 (29.2) | 171 (25.1) | 0.065 |
| Hypertension, n (%) | 473 (42.6) | 293 (43.1) | 0.831 |
| Chronic heart disease, n (%) | 192 (17.3) | 102 (15.0) | 0.206 |
| Atrial fibrillation, n (%) | 51 (4.6) | 63 (9.3) | <0.001 |
| Acute myocardial infarction, n (%) | 27 (2.4) | 12 (1.8) | 0.349 |
| Angiosclerosis, n (%) | 527 (47.4) | 445 (65.4) | <0.001 |
| Cancer, n (%) | 115 (10.4) | 143 (21.0) | <0.001 |
| Stroke, n (%) | 68 (6.1) | 88 (12.9) | <0.001 |
| Diabetes insipidus, n (%) | 12 (1.1) | 1 (0.1) | 0.049 |
| Autoimmune disease, n (%) | 83 (7.5) | 35 (5.1) | 0.054 |
| Nephrotic syndrome, n (%) | 7 (0.6) | 5 (0.7) | 1.000 |
| Inflammatory bowel disease, n (%) | 9 (0.8) | 1 (0.1) | 0.133 |
| Sepsis, n (%) | 59 (5.3) | 64 (9.4) | 0.001 |
| Thrombophilia, n (%) | 2 (0.2) | 46 (6.8) | <0.001 |
| Vital signs on admission |  |  |  |
| T (℃), Median (IQR) | 36.6 (36.5, 36.7) | 36.6 (36.5, 36.7) | 0.137 |
| RR (breaths/min), Median (IQR) | 16 (16, 18) | 16 (16, 18) | 0.872 |
| SBP (mmHg), Median (IQR) | 120 (108, 133) | 119 (107, 132) | 0.154 |
| DBP (mmHg), Median (IQR) | 66 (59, 73) | 65 (58, 72) | 0.148 |
| HR (bpm), Median (IQR) | 77 (70, 84) | 79 (72, 89) | <0.001 |
| Symptoms |  |  |  |
| Paralysis, n (%) | 4 (0.4) | 35 (5.1) | <0.001 |
| Lower limb swelling, n (%) | 32 (2.9) | 121 (17.8) | <0.001 |
| Varicose veins, n (%) | 25 (2.3) | 37 (5.4) | <0.001 |
| Pleural effusion, n (%) | 97 (8.7) | 166 (24.4) | <0.001 |
| Medication history |  |  |  |
| Glucocorticoid, n (%) | 123 (11.1) | 73 (10.7) | 0.825 |
| Contraceptive drug, n (%) | 6 (0.5) | 3 (0.4) | 1.000 |
| Dehydrant, n (%) | 141 (12.7) | 143 (21.0) | <0.001 |
| Immunosuppressive drug, n (%) | 120 (10.8) | 56 (8.2) | 0.077 |
| Surgery-related |  |  |  |
| History of surgery ≤1 month, n (%) | 312 (28.1) | 182 (26.7) | 0.545 |
| History of trauma ≤1 month, n (%) | 82 (7.4) | 193 (28.3) | <0.001 |
| Artificial instrument implantation, n (%) | 115 (10.4) | 118 (17.4) | <0.001 |
| Cardiac pacemaker, n (%) | 16 (1.4) | 15 (2.2) | 0.228 |
| General anesthesia, n (%) | 315 (28.4) | 167 (24.6) | 0.079 |
| Others |  |  |  |
| History of VTE, n (%) | 6 (0.5) | 100 (14.7) | <0.001 |
| Unexplained habitual abortion, n (%) | 5 (0.5) | 1 (0.1) | 0.512 |
| Pregnancy or puerperium, n (%) | 7 (0.6) | 8 (1.2) | 0.218 |
| Strict bed rest >3 days, n (%) | 183 (16.5) | 357 (52.5) | <0.001 |
| Central venous catheter, n (%) | 143 (12.9) | 106 (15.6) | 0.107 |
| Mechanical ventilation, n (%) | 31 (2.8) | 76 (11.2) | <0.001 |
| ICU admission, n (%) | 61 (5.5) | 142 (20.9) | <0.001 |
| Hypertonic dehydration, n (%) | 11 (1.0) | 28 (4.1) | <0.001 |
| EF, Median (IQR) | 0.66 (0.63, 0.69) | 0.65 (0.62, 0.68) | <0.001 |
| Laboratory tests |  |  |  |
| CRP, Median (IQR) | 4.47 (1.60, 29.72) | 22.84 (6.67, 65.07) | <0.001 |
| WBC, Median (IQR) | 7.05 (5.60, 9.45) | 7.90 (6.21, 10.77) | <0.001 |
| ANC, Median (IQR) | 4.69 (3.43, 6.97) | 5.79 (4.19, 8.44) | <0.001 |
| ALC, Median (IQR) | 1.50 (1.04, 2.00) | 1.26 (0.82, 1.69) | <0.001 |
| Hb, Median (IQR) | 129 (112, 142) | 116 (99, 133) | <0.001 |
| HCT, Median (IQR) | 0.38 (0.33, 0.42) | 0.35 (0.30, 0.40) | <0.001 |
| PLT, Median (IQR) | 217 (168, 266) | 204 (158, 255) | 0.004 |
| ALT, Median (IQR) | 19 (13, 31) | 20 (13, 33) | 0.071 |
| AST, Median (IQR) | 21 (16, 30) | 25 (18, 38) | <0.001 |
| TP, Median (IQR) | 66.5 (61.2, 71.4) | 63.2 (57.7, 68.4) | <0.001 |
| Alb, Median (IQR) | 40.6 (36.6, 44.0) | 36.3 (32.8, 40.1) | <0.001 |
| TBIL, Median (IQR) | 11.3 (7.9, 16.5) | 12.3 (8.7, 17.6) | 0.006 |
| Cr, Median (IQR) | 64.7 (52.8, 81.7) | 66.0 (52.9, 87.0) | 0.520 |
| Ca, Median (IQR) | 2.21 (2.10, 2.31) | 2.16 (2.05, 2.26) | <0.001 |
| TG, Median (IQR) | 1.38 (0.95, 2.10) | 1.48 (1.00, 2.24) | 0.074 |
| TC, Median (IQR) | 4.18 (3.42, 5.03) | 4.13 (3.22, 4.97) | 0.077 |
| LDL, Median (IQR) | 2.58 (1.89, 3.33) | 2.49 (1.82, 3.23) | 0.115 |
| HDL, Median (IQR) | 1 (0.8, 1.25) | 0.9 (0.71, 1.21) | <0.001 |
| D-Dimer, Median (IQR) | 0.48 (0.25, 1.42) | 4.49 (1.92, 11.12) | <0.001 |
| FIB, Median (IQR) | 3.46 (2.8, 4.42) | 3.58 (2.78, 4.76) | 0.537 |

BMI, body mass index; T, temperature; RR, respiratory rate; SBP, systolic blood pressure; DBP, diastolic blood pressure; HR, heart rate; bpm, beats per minute; VTE, venous thromboembolism; ICU, intensive care unit; IQR, interquartile range; CRP, C‑reactive protein; WBC, white blood cell; ANC, absolute neutrophil count; ALC, absolute lymphocyte count; Hb, hemoglobin; HCT, hematocrit; PLT, platelet count; ALT, alanine aminotransferase; AST, aspartate aminotransferase; TP, total protein; Alb, albumin; TBIL, total bilirubin; Cr, creatinine; Ca, calcium; TG, triglycerides; TC, total cholesterol; LDL, low‑density lipoprotein; HDL, high‑density lipoprotein; FIB, fibrinogen; EF, ejection fraction.
