# Supplementary material for: Derivation of a novel risk assessment model of venous thromboembolism in hospitalized patients
Source: PeerJ. 2026 Aug 4;14:e21598. doi: 10.7717/peerj.21598 (PMC13450040; doi:10.7717/peerj.21598)
Supplement: Supplementary file 13 [file peerj-14-21598-s013.docx]

Table S2 Multivariate regression analysis for thromboembolism events (data-driven)

| Variable | OR (95%CI) | *p*-value | coefficients |
| --- | --- | --- | --- |
| Age, years |  |  |  |
| ≤30 | - | - | - |
| >30 to ≤40 | 1.03 (0.32-3.43) | 0.959 | 0.031 |
| >40 to ≤50 | 0.99 (0.36-2.98) | 0.987 | -0.009 |
| >50 to ≤60 | 2.10 (0.80-6.05) | 0.150 | 0.740 |
| >60 to ≤70 | 2.62 (1.02-7.48) | 0.057 | 0.965 |
| >70 | 3.27 (1.28-9.23) | 0.018 | 1.185 |
| Cancer | 2.15 (1.50-3.10) | < 0.001 | 0.768 |
| Stroke | 2.35 (1.50-3.70) | < 0.001 | 0.856 |
| Thrombophilia | 41.54 (10.83-279.29) | < 0.001 | 3.727 |
| Heart rate >100 bpm | 4.03 (2.18-7.69) | < 0.001 | 1.394 |
| Paralysis | 3.73 (1.34-13.31) | 0.021 | 1.316 |
| Lower limb swelling | 4.60 (2.84-7.63) | < 0.001 | 1.527 |
| Varicose veins | 3.68 (1.74-7.91) | <0.001 | 1.302 |
| Pleural effusion | 1.72 (1.20-2.47) | 0.003 | 0.541 |
| History of trauma ≤1 month | 4.77 (3.30-6.95) | < 0.001 | 1.562 |
| Artificial instrument implantation | 1.18 (0.82-1.71) | 0.367 | 0.169 |
| History of VTE | 33.88 (14.32-96.11) | < 0.001 | 3.523 |
| Pregnancy or puerperium | 0.31 (0.01-2.62) | 0.346 | -1.181 |
| Strict bed rest >3 days | 2.16 (1.59-2.94) | < 0.001 | 0.771 |
| Hypertonic dehydration | 1.50 (0.63-3.80) | 0.373 | 0.405 |
| ICU admission | 1.40 (0.92-2.13) | 0.115 | 0.336 |
| Alb <40 g/L | 1.41 (1.06-1.88) | 0.019 | 0.343 |
| TG >1.7 mmol/L | 7.92 (5.20-12.43) | < 0.001 | 2.069 |
| D-Dimer >0.5 μg/mL | 1.41 (1.07-1.86) | 0.015 | 0.343 |

Only variables with *P* < 0.05 after LASSO selection were retained. Non‑significant variables (artificial instrument implantation, pregnancy or puerperium, hypertonic dehydration, ICU admission) were excluded.

OR, odds ratio; CI, confidence interval; VTE, venous thromboembolism; Alb, albumin; TG, triglycerides; bpm, beats per minute; ICU, Intensive care unit.
