# Supplementary material for: Derivation of a novel risk assessment model of venous thromboembolism in hospitalized patients
Source: PeerJ. 2026 Aug 4;14:e21598. doi: 10.7717/peerj.21598 (PMC13450040; doi:10.7717/peerj.21598)
Supplement: Supplementary file 14 [file peerj-14-21598-s014.docx]

Table S3 Performance comparison of six scores for predicting VTE (n=1791)

| Score | AUROC | SEN | SPE | PLR | NLR | PPV | NPV |
| --- | --- | --- | --- | --- | --- | --- | --- |
| Caprini Score | 0.698 (0.674-0.723) | 0.743 (0.710-0.776) | 0.581 (0.552-0.610) | 1.774 (1.634-1.926) | 0.443 (0.386-0.508) | 0.521 (0.489-0.552) | 0.787 (0.759-0.815) |
| Padua Score | 0.786 (0.765-0.808) | 0.700 (0.666-0.734) | 0.788 (0.764-0.812) | 3.309 (2.924-3.745) | 0.380 (0.338-0.428) | 0.669 (0.635-0.704) | 0.811 (0.788-0.834) |
| Wells Score | 0.687 (0.663-0.710) | 0.787 (0.756-0.818) | 0.501 (0.472-0.531) | 1.578 (1.470-1.693) | 0.425 (0.364-0.497) | 0.491 (0.462-0.521) | 0.793 (0.763-0.823) |
| Geneva Score | 0.716 (0.692-0.739) | 0.772 (0.741-0.804) | 0.546 (0.517-0.576) | 1.702 (1.577-1.837) | 0.417 (0.360-0.484) | 0.510 (0.480-0.541) | 0.797 (0.768-0.825) |
| Autar Score | 0.789 (0.768-0.810) | 0.816 (0.787-0.845) | 0.633 (0.604-0.661) | 2.222 (2.041-2.420) | 0.291 (0.246-0.342) | 0.576 (0.545-0.608) | 0.849 (0.825-0.873) |
| Weng Score | 0.895 (0.880-0.909) | 0.843 (0.815-0.870) | 0.783 (0.759-0.807) | 3.885 (3.458-4.364) | 0.201 (0.168-0.240) | 0.704 (0.673-0.735) | 0.890 (0.871-0.910) |

VTE, venous thromboembolism; AUROC, area under the receiver operating characteristic curve; SEN, sensitivity; SPE, specificity; PLR, positive likelihood ratio; NLR, negative likelihood ratio; PPV, positive predictive value; NPV, negative predictive value.

The analysis for thromboembolism prediction included all VTE events (deep vein thrombosis and pulmonary embolism, n = 680) and non‑VTE controls (n = 1,111), total n = 1,791.
