# Supplementary material for: Derivation of a novel risk assessment model of venous thromboembolism in hospitalized patients
Source: PeerJ. 2026 Aug 4;14:e21598. doi: 10.7717/peerj.21598 (PMC13450040; doi:10.7717/peerj.21598)
Supplement: Supplementary file 15 [file peerj-14-21598-s015.docx]

Table S4 Performance comparison of six scores for predicting VTE in internal medicine population (n=1070)

| Score | AUROC | SEN | SPE | PLR | NLR | PPV | NPV |
| --- | --- | --- | --- | --- | --- | --- | --- |
| Caprini Score | 0.717 (0.685-0.749) | 0.710 (0.663-0.758) | 0.673 (0.638-0.707) | 2.170 (1.916-2.457) | 0.431 (0.363-0.511) | 0.515 (0.471-0.560) | 0.826 (0.795-0.856) |
| Padua Score | 0.808 (0.780-0.835) | 0.776 (0.732-0.819) | 0.727 (0.694-0.760) | 2.841 (2.490-3.242) | 0.309 (0.253-0.377) | 0.582 (0.537-0.627) | 0.869 (0.842-0.896) |
| Wells Score | 0.746 (0.716-0.775) | 0.761 (0.717-0.806) | 0.673 (0.638-0.707) | 2.326 (2.063-2.623) | 0.355 (0.292-0.430) | 0.533 (0.489-0.576) | 0.852 (0.823-0.881) |
| Geneva Score | 0.748 (0.719-0.777) | 0.781 (0.738-0.824) | 0.617 (0.581-0.653) | 2.040 (1.831-2.273) | 0.355 (0.289-0.436) | 0.500 (0.458-0.542) | 0.852 (0.821-0.882) |
| Autar Score | 0.808 (0.781-0.835) | 0.827 (0.787-0.866) | 0.655 (0.620-0.689) | 2.393 (2.141-2.676) | 0.265 (0.209-0.335) | 0.540 (0.498-0.582) | 0.885 (0.858-0.912) |
| Weng Score | 0.900 (0.881-0.918) | 0.824 (0.784-0.864) | 0.799 (0.770-0.829) | 4.108 (3.522-4.791) | 0.220 (0.175-0.277) | 0.668 (0.624-0.713) | 0.903 (0.879-0.926) |

VTE, venous thromboembolism; AUROC, area under the receiver operating characteristic curve; SEN, sensitivity; SPE, specificity; PLR, positive likelihood ratio; NLR, negative likelihood ratio; PPV, positive predictive value; NPV, negative predictive value.

The analysis for thromboembolism prediction in the internal medicine population included all VTE events (n = 352) and non‑VTE controls (n = 718), total n = 1,070.
