# Supplementary material for: Derivation of a novel risk assessment model of venous thromboembolism in hospitalized patients
Source: PeerJ. 2026 Aug 4;14:e21598. doi: 10.7717/peerj.21598 (PMC13450040; doi:10.7717/peerj.21598)
Supplement: Supplementary file 16 [file peerj-14-21598-s016.docx]

Table S5 Performance comparison of six scores for predicting VTE in surgical population (n=721)

| Score | AUROC | SEN | SPE | PLR | NLR | PPV | NPV |
| --- | --- | --- | --- | --- | --- | --- | --- |
| Caprini Score | 0.654 (0.614-0.694) | 0.515 (0.461-0.569) | 0.753 (0.711-0.796) | 2.088 (1.706-2.555) | 0.644 (0.568-0.729) | 0.635 (0.577-0.693) | 0.651 (0.607-0.694) |
| Padua Score | 0.745 (0.708-0.782) | 0.726 (0.677-0.774) | 0.738 (0.694-0.781) | 2.769 (2.315-3.310) | 0.372 (0.309-0.448) | 0.698 (0.649-0.747) | 0.763 (0.720-0.806) |
| Wells Score | 0.569 (0.532-0.606) | 0.284 (0.235-0.332) | 0.875 (0.843-0.908) | 2.274 (1.662-3.111) | 0.819 (0.757-0.885) | 0.655 (0.577-0.733) | 0.594 (0.554-0.634) |
| Geneva Score | 0.658 (0.620-0.696) | 0.460 (0.406-0.514) | 0.809 (0.770-0.848) | 2.412 (1.907-3.051) | 0.667 (0.597-0.745) | 0.668 (0.607-0.730) | 0.642 (0.600-0.685) |
| Autar Score | 0.745 (0.709-0.780) | 0.872 (0.836-0.908) | 0.486 (0.437-0.535) | 1.696 (1.528-1.884) | 0.263 (0.195-0.356) | 0.586 (0.542-0.630) | 0.820 (0.770-0.869) |
| Weng Score | 0.885 (0.861-0.909) | 0.851 (0.812-0.889) | 0.771 (0.729-0.813) | 3.714 (3.081-4.478) | 0.194 (0.149-0.252) | 0.756 (0.712-0.800) | 0.861 (0.825-0.897) |

VTE, venous thromboembolism; AUROC, area under the receiver operating characteristic curve; SEN, sensitivity; SPE, specificity; PLR, positive likelihood ratio; NLR, negative likelihood ratio; PPV, positive predictive value; NPV, negative predictive value.

The analysis for thromboembolism prediction in the surgical population included all VTE events (n = 328) and non‑VTE controls (n = 393), total n = 721.
