# Supplementary material for: Derivation of a novel risk assessment model of venous thromboembolism in hospitalized patients
Source: PeerJ. 2026 Aug 4;14:e21598. doi: 10.7717/peerj.21598 (PMC13450040; doi:10.7717/peerj.21598)
Supplement: Supplementary file 17 [file peerj-14-21598-s017.docx]

Table S6 Performance comparison of six scores for predicting PE (n=1274)

| Score | AUROC | SEN | SPE | PLR | NLR | PPV | NPV |
| --- | --- | --- | --- | --- | --- | --- | --- |
| Caprini Score | 0.682 (0.640-0.725) | 0.485 (0.408-0.561) | 0.811 (0.788-0.834) | 2.564 (2.100-3.131) | 0.635 (0.546-0.739) | 0.273 (0.222-0.325) | 0.915 (0.897-0.932) |
| Padua Score | 0.769 (0.729-0.809) | 0.650 (0.577-0.724) | 0.788 (0.764-0.812) | 3.074 (2.620-3.607) | 0.444 (0.359-0.548) | 0.311 (0.262-0.360) | 0.939 (0.924-0.954) |
| Wells Score | 0.743 (0.701-0.786) | 0.466 (0.390-0.543) | 0.925 (0.910-0.941) | 6.241 (4.792-8.128) | 0.577 (0.499-0.666) | 0.478 (0.400-0.556) | 0.922 (0.906-0.938) |
| Geneva Score | 0.682 (0.642-0.721) | 0.755 (0.689-0.821) | 0.546 (0.517-0.576) | 1.663 (1.492-1.855) | 0.449 (0.341-0.591) | 0.196 (0.165-0.227) | 0.938 (0.920-0.957) |
| Autar Score | 0.761 (0.721-0.800) | 0.761 (0.695-0.826) | 0.633 (0.604-0.661) | 2.072 (1.845-2.325) | 0.378 (0.287-0.499) | 0.233 (0.197-0.269) | 0.947 (0.931-0.963) |
| Weng Score | 0.877 (0.851-0.903) | 0.859 (0.805-0.912) | 0.700 (0.673-0.727) | 2.866 (2.569-3.197) | 0.201 (0.138-0.295) | 0.296 (0.255-0.337) | 0.971 (0.960-0.983) |

AUROC, area under the receiver operating characteristic curve; SEN, sensitivity; SPE, specificity; PLR, positive likelihood ratio; NLR, negative likelihood ratio; PPV, positive predictive value; NPV, negative predictive value.

For PE prediction, the analysis included 163 PE cases and 1,111 non‑VTE controls (total n = 1,274); patients with DVT only (n = 517) were excluded.
