# Supplementary material for: Derivation of a novel risk assessment model of venous thromboembolism in hospitalized patients
Source: PeerJ. 2026 Aug 4;14:e21598. doi: 10.7717/peerj.21598 (PMC13450040; doi:10.7717/peerj.21598)
Supplement: Supplementary file 18 [file peerj-14-21598-s018.docx]

Table S7 Performance comparison of six scores for predicting PE in internal medicine population (n=842)

| Score | AUROC | SEN | SPE | PLR | NLR | PPV | NPV |
| --- | --- | --- | --- | --- | --- | --- | --- |
| Caprini Score | 0.699 (0.651-0.747) | 0.661 (0.578-0.745) | 0.673 (0.638-0.707) | 2.020 (1.715-2.380) | 0.504 (0.392-0.647) | 0.259 (0.210-0.307) | 0.920 (0.897-0.943) |
| Padua Score | 0.786 (0.743-0.830) | 0.726 (0.647-0.804) | 0.727 (0.694-0.760) | 2.659 (2.263-3.124) | 0.377 (0.282-0.504) | 0.315 (0.261-0.369) | 0.939 (0.919-0.959) |
| Wells Score | 0.779 (0.734-0.824) | 0.782 (0.710-0.855) | 0.673 (0.638-0.707) | 2.390 (2.078-2.749) | 0.324 (0.231-0.454) | 0.292 (0.243-0.341) | 0.947 (0.928-0.966) |
| Geneva Score | 0.721 (0.677-0.764) | 0.750 (0.674-0.826) | 0.617 (0.581-0.653) | 1.958 (1.706-2.247) | 0.405 (0.297-0.553) | 0.253 (0.208-0.297) | 0.935 (0.912-0.957) |
| Autar Score | 0.769 (0.725-0.813) | 0.774 (0.701-0.848) | 0.655 (0.620-0.689) | 2.241 (1.952-2.574) | 0.345 (0.248-0.480) | 0.279 (0.232-0.326) | 0.944 (0.924-0.964) |
| Weng Score | 0.890 (0.862-0.918) | 0.839 (0.774-0.903) | 0.747 (0.715-0.778) | 3.309 (2.855-3.834) | 0.216 (0.144-0.323) | 0.364 (0.308-0.419) | 0.964 (0.949-0.980) |

AUROC, area under the receiver operating characteristic curve; SEN, sensitivity; SPE, specificity; PLR, positive likelihood ratio; NLR, negative likelihood ratio; PPV, positive predictive value; NPV, negative predictive value, PE, pulmonary embolism.

For PE prediction in the internal medicine population, the analysis included 124 PE cases and 718 non‑VTE controls (total n = 842). Patients with deep vein thrombosis (DVT) only were excluded from the PE‑specific analyses.
