# Supplementary material for: Derivation of a novel risk assessment model of venous thromboembolism in hospitalized patients
Source: PeerJ. 2026 Aug 4;14:e21598. doi: 10.7717/peerj.21598 (PMC13450040; doi:10.7717/peerj.21598)
Supplement: Supplementary file 19 [file peerj-14-21598-s019.docx]

Table S8 Performance comparison of six scores for predicting PE in surgical population (n=432)

| Score | AUROC | SEN | SPE | PLR | NLR | PPV | NPV |
| --- | --- | --- | --- | --- | --- | --- | --- |
| Caprini Score | 0.730 (0.642-0.818) | 0.692 (0.547-0.837) | 0.753 (0.711-0.796) | 2.805 (2.138-3.679) | 0.409 (0.254-0.656) | 0.218 (0.145-0.290) | 0.961 (0.939-0.983) |
| Padua Score | 0.792 (0.718-0.867) | 0.821 (0.700-0.941) | 0.738 (0.694-0.781) | 3.131 (2.509-3.907) | 0.243 (0.124-0.477) | 0.237 (0.165-0.309) | 0.976 (0.959-0.994) |
| Wells Score | 0.743 (0.643-0.843) | 0.615 (0.463-0.768) | 0.875 (0.843-0.908) | 4.936 (3.441-7.080) | 0.439 (0.295-0.655) | 0.329 (0.221-0.437) | 0.958 (0.938-0.979) |
| Geneva Score | 0.610 (0.523-0.696) | 0.769 (0.637-0.901) | 0.417 (0.369-0.466) | 1.320 (1.090-1.598) | 0.553 (0.308-0.992) | 0.116 (0.077-0.155) | 0.948 (0.915-0.981) |
| Autar Score | 0.841 (0.772-0.910) | 0.744 (0.607-0.881) | 0.781 (0.740-0.822) | 3.398 (2.614-4.418) | 0.328 (0.192-0.562) | 0.252 (0.173-0.332) | 0.968 (0.949-0.988) |
| Weng Score | 0.850 (0.790-0.910) | 0.769 (0.637-0.901) | 0.771 (0.729-0.813) | 3.359 (2.616-4.313) | 0.299 (0.168-0.532) | 0.250 (0.173-0.327) | 0.971 (0.953-0.990) |

AUROC, area under the receiver operating characteristic curve; SEN, sensitivity; SPE, specificity; PLR, positive likelihood ratio; NLR, negative likelihood ratio; PPV, positive predictive value; NPV, negative predictive value; PE, pulmonary embolism.

For PE prediction in the surgical population, the analysis included 39 PE cases and 393 non‑VTE controls (total n = 432). Patients with DVT only were excluded from the PE‑specific analyses.
