# Supplementary material for: Derivation of a novel risk assessment model of venous thromboembolism in hospitalized patients
Source: PeerJ. 2026 Aug 4;14:e21598. doi: 10.7717/peerj.21598 (PMC13450040; doi:10.7717/peerj.21598)
Supplement: Supplementary file 20 [file peerj-14-21598-s021.docx]

| **Variable Name** | **Description** | **Data Type** | **Coding / Values** | **Notes** |
| --- | --- | --- | --- | --- |
| order | Record ID (sequential number) | Numeric (integer) | 1–1792 | Order |
| data | Date of admission or data collection | Date | Excel date format | — |
| age | Patient age (years) | Numeric (continuous) | 18–94 | — |
| male | Gender | Text | “man” / “woman” | — |
| BMI | Body mass index (kg/m²) | Numeric (continuous) | 11.02–36.51 | — |
| smoking | Current or former smoking status | Text | “yes” / “no” | — |
| drinking | Current or former alcohol consumption | Text | “yes” / “no” | — |
| embolism | History of any embolism | Text | “yes” / “no” | — |
| Diabetes mellitus | Diagnosis of diabetes mellitus | Text | “yes” / “no” | — |
| hypertension | Diagnosis of hypertension | Text | “yes” / “no” | — |
| chronic heart disease | Chronic heart disease | Text | “yes” / “no” | — |
| atrial fibrillation | Atrial fibrillation | Text | “yes” / “no” | — |
| acute myocardial infarction | Acute myocardial infarction | Text | “yes” / “no” | — |
| angiosclerosis | Angiosclerosis / arteriosclerosis | Text | “yes” / “no” | — |
| cancer | Active or history of cancer | Text | “yes” / “no” | — |
| diabetes insipidus | Diabetes insipidus | Text | “yes” / “no” | — |
| sepsis | Sepsis | Text | “yes” / “no” | — |
| nephrotic syndrome | Nephrotic syndrome | Text | “yes” / “no” | — |
| inflammatory bowel disease | Inflammatory bowel disease | Text | “yes” / “no” | — |
| autoimmune disease | Autoimmune disease | Text | “yes” / “no” | — |
| stroke | History of stroke | Text | “yes” / “no” | — |
| thrombophilia | Thrombophilia (inherited or acquired) | Text | “yes” / “no” | — |
| Temperature | Body temperature (°C) | Numeric (continuous) | 36.0–38.2 | — |
| respiratory rates | Respiratory rate (breaths/min) | Numeric (integer) | 15–25 | — |
| systolic blood pressure | Systolic blood pressure (mmHg) | Numeric (integer) | 91–155 | — |
| diastolic blood pressure | Diastolic blood pressure (mmHg) | Numeric (integer) | 46–99 | — |
| heart rate | Heart rate (beats/min) | Numeric (integer) | 54–120 | — |
| Paralysis | Paralysis (any) | Text | “yes” / “no” | — |
| lower limb swelling | Lower limb swelling | Text | “yes” / “no” | — |
| varicose veins | Varicose veins | Text | “yes” / “no” | — |
| Glucocorticoid | Glucocorticoid use | Text | “yes” / “no” | — |
| contraceptive drug | Contraceptive drug use | Text | “yes” / “no” | — |
| dehydrant | Dehydrant use | Text | “yes” / “no” | — |
| pleural effusion | Pleural effusion | Text | “yes” / “no” | — |
| Surgery | Recent surgery | Text | “yes” / “no” | — |
| artificial instrument implantation | Artificial instrument implantation | Text | “yes” / “no” | — |
| cardiac pacemaker | Cardiac pacemaker implantation | Text | “yes” / “no” | — |
| trauma | Recent trauma | Text | “yes” / “no” | — |
| general anesthesia | General anesthesia | Text | “yes” / “no” | — |
| Personal or family history of VTE | Personal or family history of VTE | Text | “yes” / “no” | — |
| unexplained habitual abortion | Unexplained habitual abortion | Text | “yes” / “no” | — |
| pregnancy or puerperium | Pregnancy or puerperium | Text | “yes” / “no” | — |
| strict bed rest | Strict bed rest (>3 days) | Text | “yes” / “no” | — |
| central venous catheter | Central venous catheter | Text | “yes” / “no” | — |
| mechanical ventilation | Mechanical ventilation | Text | “yes” / “no” | — |
| hypertonic dehydration | Hypertonic dehydration | Text | “yes” / “no” | — |
| ICU admission | ICU admission | Text | “yes” / “no” | — |
| ef | Ejection fraction (%) | Numeric (continuous) | 0.27–0.78 | — |
| CRP | C-reactive protein (mg/L) | Numeric (continuous) | 0–344.36 | — |
| WBC | White blood cell count (×10⁹/L) | Numeric (continuous) | 3.19–495.25 | — |
| ANC | Absolute neutrophil count (×10⁹/L) | Numeric (continuous) | 0.726–242.673 | — |
| ALC | Absolute lymphocyte count (×10⁹/L) | Numeric (continuous) | 0.145–19.686 | — |
| HCT | Hematocrit (%) | Numeric (continuous) | 0.095–0.585 | — |
| HB | Hemoglobin (g/L) | Numeric (continuous) | 44–220 | — |
| PLT | Platelet count (×10⁹/L) | Numeric (continuous) | 11–887 | — |
| ALT | Alanine aminotransferase (U/L) | Numeric (continuous) | 4–413 | — |
| AST | Aspartate aminotransferase (U/L) | Numeric (continuous) | 6–499 | — |
| TP | Total protein (g/L) | Numeric (continuous) | 44.7–83.4 | — |
| Alb | Albumin (g/L) | Numeric (continuous) | 21.9–51.1 | — |
| TBIL | Total bilirubin (μmol/L) | Numeric (continuous) | 2.5–59.6 | — |
| Cr | Creatinine (μmol/L) | Numeric (continuous) | 34.6–734 | — |
| Ca | Calcium (mmol/L) | Numeric (continuous) | 1.43–4.12 | — |
| TG | Triglycerides (mmol/L) | Numeric (continuous) | 0.07–5.85 | — |
| TC | Total cholesterol (mmol/L) | Numeric (continuous) | 0.68–16.01 | — |
| LDL | Low-density lipoprotein (mmol/L) | Numeric (continuous) | 0.39–5.59 | — |
| HDL | High-density lipoprotein (mmol/L) | Numeric (continuous) | 0.22–2.25 | — |
| D-Dimer | D-dimer (mg/L FEU) | Numeric (continuous) | 0.22–57.5 | — |
| FIB | Fibrinogen (g/L) | Numeric (continuous) | 0.6–20 | — |
| immunosuppressive drug | Immunosuppressive drug use | Text | “yes” / “no” | — |
| OUTCOME | Clinical outcome (VTE event) | Text | “no”, “deep vein thrombosis”, “pulmonary embolism”, “Arterial Embolism” | — |
| **Caprini Score** | **Caprini VTE risk assessment total score** | **Numeric (ordinal)** | **0–25** | Raw total score from validated Caprini model |
| **Padua Score** | **Padua VTE risk assessment total score** | **Numeric (ordinal)** | **0–11** | Raw total score from validated Padua model |
| **Wells Score** | **Wells score for DVT/PE probability** | **Numeric (ordinal)** | **0–5** | Raw total score from validated Wells model |
| **Geneva Score** | **Revised Geneva score for PE probability** | **Numeric (ordinal)** | **0–5** | Raw total score from validated Geneva model |
| **Autar Score** | **Autar VTE risk assessment total score** | **Numeric (ordinal)** | **1–25** | Raw total score from validated Autar model |
