# Supplementary material for: Derivation of a novel risk assessment model of venous thromboembolism in hospitalized patients
Source: PeerJ. 2026 Aug 4;14:e21598. doi: 10.7717/peerj.21598 (PMC13450040; doi:10.7717/peerj.21598)
Supplement: Supplementary file 21 [file peerj-14-21598-s022.docx]

**Clear explanation of the analytic population for PE prediction**

The total study cohort comprised 1,791 hospitalized patients, including 680 patients with any venous thromboembolism (VTE) event and 1,111 patients without VTE. Among the 680 VTE patients, 163 had pulmonary embolism (PE) (with or without concomitant deep vein thrombosis [DVT]), and the remaining 517 had DVT alone.

For the **VTE prediction analyses**, all 680 VTE events (both DVT and PE) were used as cases, with the 1,111 non‑VTE patients as controls, resulting in a total sample of 1,791 patients.

For the **PE‑specific prediction analyses**, only the 163 patients with PE were used as cases. The control group remained the same 1,111 patients without any VTE. Patients with DVT alone (n = 517) were excluded from the PE analyses to avoid outcome misclassification and to maintain a clear case definition for PE. Thus, the PE prediction analyses were based on a sample of **1,274 patients (163 PE cases + 1,111 non‑VTE controls)**.

**Subgroup breakdown for PE prediction:**

- **Internal medicine population**: 124 PE cases and 718 non‑VTE controls (total n = 842).
- **Surgical population**: 39 PE cases and 393 non‑VTE controls (total n = 432).

These definitions are consistently applied in Table 5, Table 6 (PE section), and supplementary Tables S6–S8, each of which includes a footnote stating the exact sample sizes. The predicted probabilities for PE derived from this case‑control sampled cohort reflect relative risk ordering; external validation in a representative cohort is required to obtain absolute risk estimates.
