# Supplementary material for: Derivation of a novel risk assessment model of venous thromboembolism in hospitalized patients
Source: PeerJ. 2026 Aug 4;14:e21598. doi: 10.7717/peerj.21598 (PMC13450040; doi:10.7717/peerj.21598)
Supplement: Supplementary file 22 [file peerj-14-21598-s023.docx]

**Corrected and reproducible Weng score derivation**

The Weng score was derived from the clinical knowledge‑driven model (Model A) using multivariable logistic regression. The following steps ensure full reproducibility:

**Step 1 – Regression coefficients**

The final model included 14 independent predictors (Table 3). The regression coefficient (β) for each variable was obtained from the multivariable logistic regression model.

**Step 2 – Identify the smallest coefficient**

The smallest absolute β value among all predictors was **0.301**, corresponding to Albumin <40 g/L.

**Step 3 – Calculate raw points**

For each variable, the raw point value was calculated as:
Raw points = β / 0.301

**Step 4 – Round to integer or half‑integer**

Raw points were rounded to the nearest 0.5 to create clinically usable integer or half‑integer scores. Examples:

- VTE history: 3.500 / 0.301 = 11.63 → rounded to 11.5
- D‑Dimer >0.5 μg/mL: 2.111 / 0.301 = 7.01 → rounded to 7.0
- Age >70 years: 1.313 / 0.301 = 4.36 → rounded to 4.5
- Albumin <40 g/L: 0.301 / 0.301 = 1.00 → 1.0

**Step 5 – Total score**

The total Weng score for a patient is the sum of points for all present risk factors. The theoretical range is **0 to 70.5**.

**Step 6 – Risk stratification**

Based on the distribution of total scores in the derivation cohort, four risk categories were defined:

- Low risk: 0–4 points
- Moderate risk: >4 to ≤10 points
- High risk: >10 to ≤14 points
- Very high risk: >14 points

**Step 7 – Sample‑based risk estimates**

For each risk stratum, the predicted probability of VTE (or PE) was obtained from the logistic regression model. These sample‑based estimates are presented in Table 6 with the explicit caveat that they are conditional on the case‑control sampling design and are intended for relative risk stratification, not direct generalization as absolute population risks.

**Reproducibility note**

A clinician can calculate the Weng score using the paper‑based checklist in Table 3: simply add the points for each risk factor present. No electronic calculator or regression equation is needed. The derivation is fully transparent and can be replicated from the information provided in Table 3 and its footnote.
